# Novel Tetraene Macrodiolides Are Effective Inducers of Mitochondrial Apoptosis in Jurkat Cells

**DOI:** 10.3390/ijms26115139

**Published:** 2025-05-27

**Authors:** Ilgiz I. Islamov, Lilya U. Dzhemileva, Ilgam V. Gaisin, Alexey A. Makarov, Usein M. Dzhemilev, Vladimir A. D’yakonov

**Affiliations:** 1 Institute of Petrochemistry and Catalysis, Ufa Federal Research Centre, Russian Academy of Sciences, 141 Prospekt Oktyabrya, Ufa 450075, Russia; 2 N.D. Zelinsky Institute of Organic Chemistry, Russian Academy of Sciences, Leninsky Prospect 47, Moscow 119991, Russia

**Keywords:** homo-cyclomagnesiation, 1,5-dienoic compounds, cyclocondensation, tetraenes, macrodiolides, apoptosis, antitumor agents

## Abstract

We synthesized 16 representatives of a new class of tetraene macrodiolides with two pharmacophore *cis*,*cis*-1,5-diene fragments of the molecule in their structure in rather high yields (from 67 to 84%), which, in turn, were synthesized by a catalytic intermolecular cyclocondensation reaction of α,ω-alka-n*Z*,(n+4)*Z*-diendiols with α,ω-alka-n*Z*,(n+4)*Z*-diendioic acids using Hf(OTf)_4_. The synthesis of starting substrates with 1*Z*,5*Z*-diene moieties with a high degree of stereoselectivity was carried out using the authors’ original reaction of catalytic homo-cyclomagnesiation of O-containing allenes. The cytotoxic potential of the examined compounds was assessed using the following cell lines: Jurkat, K562, U937, HL60, HEK293, and Wi-38 (fibroblasts). Biological tests of the synthesized compounds showed a direct effect on mitochondrial biogenesis by the dissociation of oxidation and phosphorylation and the release of cytochrome P450 into the cell cytosol, as well as the induction of mitochondrial apoptosis. The selectivity index demonstrates significant variability, ranging from approximately 2.5 to 5.3 for Jurkat cells and from 3.0 to 5.8 for the other cell lines.

## 1. Introduction

**Figure 1:**
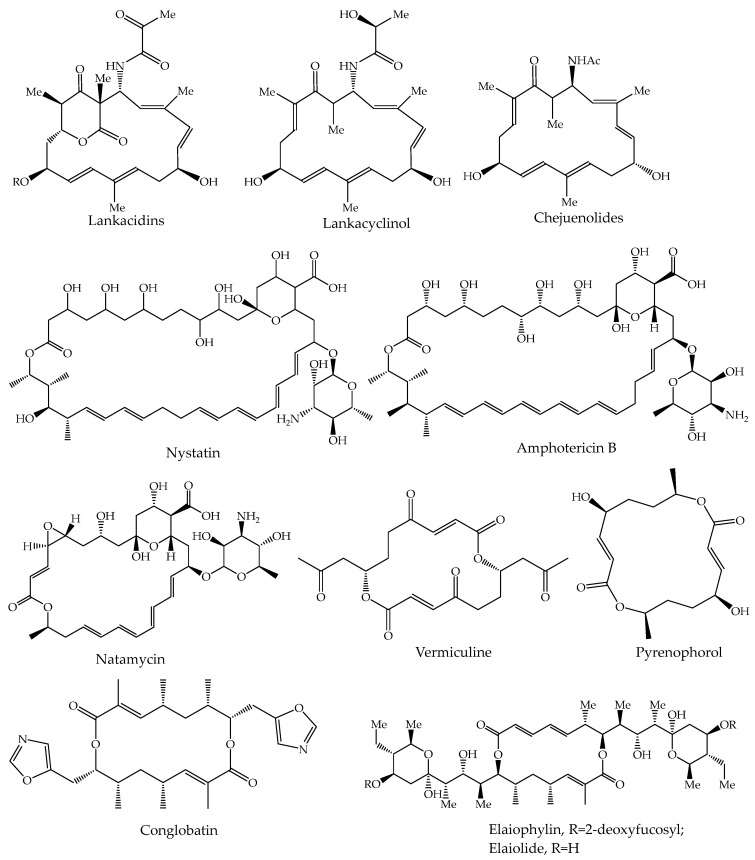
Structures of some natural macrocycles.

Macrocycles represent a vast class of compounds that possess distinctive physicochemical and biomedical properties. These compounds occur in nature and are utilized by humans in numerous fields, including supramolecular chemistry, catalysis, materials science, and pharmaceuticals. In the field of chemistry, macrocycles frequently demonstrate the capacity to function as ligands, thereby conferring remarkable thermodynamic and kinetic stability to transition metal complexes (Figure 1) [1,2,3,4,5,6,7,8]. The quest for novel methodologies to modulate novel drug targets has precipitated a surge of interest in new therapeutic methods among biopharmaceutical companies and drug development scientists. Macrocycles and cyclic peptides exemplify the recent advancements witnessed in the chemical and pharmaceutical industries, respectively. Over the past decade, there has been a substantial increase in the number of FDA-approved macrocyclic drugs, as well as in the development of clinical drug candidates in this category [9].

For example, the natural tetraene macrocarbocycles Chejuenolides and Lankacidins are of considerable interest as the basis for the development of a new generation of medical drugs [10,11,12]. The discovery of Chejuenolides A, B, and C, the 17-membered carbocyclic tetraene natural products from the culture extract of the marine bacterium Hahella chejuensis MB-1084, isolated from marine sediments on the shelf of Geoje Island, represents a significant advancement in the field [10]. Chejuenolides have been shown to inhibit protein tyrosine phosphatase 1B (PTP1B), which is an important target in the development of drugs for the treatment of type 2 diabetes and related metabolic syndrome [13,14]. The antibacterial effect of compounds consisting of 17-membered carbocyclic rings has been previously reported for Lancacidin, an antibacterial drug used to treat Serpulina (Treponema) hyodysenteriae infection and spirochetosis in livestock. Lancacidin has been demonstrated to possess both anticancer and immunosuppressive properties. These compounds have demonstrated antitumor activity in vitro against leukemia L1210, melanoma B16, and lymphosarcoma 6C3 HED/OG, exhibiting low toxicity and good tolerability in vivo when administered orally to mice [15,16].

Polyene macrolides, which are 26–38-membered macrocycles with conjugated double bonds, merit particular consideration and have found wide application in medicine as antibiotics, antiparasitic agents, and antifungal agents [17,18,19,20]. Specifically, nystatin is employed in the management of mucosal infections caused by Candida albicans [21]. Natamycin (pimaricin) is employed in the treatment of ocular infections [22]. Amphotericin B is regarded as the gold standard for the treatment of systemic mycoses and is also employed against diseases caused by Leishmania [23].

It has been demonstrated that naturally occurring bioactive macrodiolides [24,25,26], including pyrenophorol [27,28,29], bispolides [30], and vermiculin [31], exhibit pronounced antibacterial properties. Conglobatins, on the other hand, have been shown to be Hsp90 inhibitors with antitumor activity [32]. Elaiophylins represent a distinguished class of tetraene macrodiolides that exhibit a wide spectrum of pharmacological properties, encompassing antimicrobial, antihelminthic, immunodepressant, anti-inflammatory, and antiviral activities. This macrodiolide has been demonstrated to elicit anticancer effects by impeding autophagy in its late stages, a property that holds particular promise for the treatment of ovarian cancer, multiple myeloma, and lung adenocarcinoma [33,34].

The preceding illustrations demonstrate that unsaturated macrocyclic compounds represent a pivotal foundation in the formulation of fungicidal, antibacterial, and antitumor pharmaceuticals. Consequently, their pharmaceutical applications are undergoing rapid expansion. Concurrently, the emergence of adverse effects and the evolution of drug resistance perpetually impel researchers to explore more efficacious compounds with enhanced pharmacological properties.

Over the past decade, our research group has been engaged in synthesizing and studying the biological properties of natural and synthetic compounds (fatty acids, acetogenins, lembehynes, hybrid molecules) containing a pharmacophore *cis*,*cis*-1,5-diene fragment in their structure [35,36]. In consideration of the significant pharmacological potential of macrocycles, a research initiative has been undertaken to develop novel methodologies for the synthesis of 1Z,5Z-diene macrodiolides. This endeavor represents a pioneering advancement in the field, as it is the first time such methods have been employed. The presence of bis-methylene-interrupted Z-double bonds has been demonstrated to result in the observed antitumor effect of synthesized macrolactones [37]. The antitumor activity of the macrodiolides in vitro against tumor cell lines (Jurkat, K562, U937, HL-60, and HeLa) has been demonstrated. These macrodiolides have been shown to induce mitochondrial apoptosis of immortalized T-cells (Jurkat), effectively inhibit the phosphorylation of Akt, p38 kinases, and the transcription factor CREB in tumor cells, and inhibit the process of cell division [38,39,40]. The objective of the present study is to synthesize new tetraene macrodiolides containing two 1*Z*,5*Z*-diene fragments in their structure and to study the anticancer properties of these compounds, including in comparison with previously synthesized diene macrocycles.

## 2. Results and Discussion

### 2.1. Chemistry

**Scheme 1:**
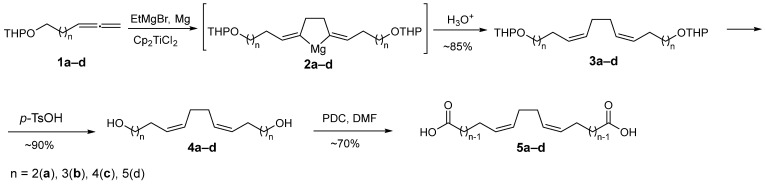
Synthesis of α,ω-alca-n*Z*,(n+4)*Z*-dienediols **4a**–**d** and α,ω-alca-n*Z*,(n+4)*Z*-dienedioic acids **5a**–**d**.

As previously mentioned, a high antitumor potential was identified for the synthesized unsaturated *cis*,*cis*-1,5-diene macrodiolides. However, the possibility of synthesizing macrocyclic compounds containing multiple diene fragments, as well as the impact of these groups on the antitumor properties of the obtained macrodiolides, remained to be elucidated. For the first time, we synthesized similar tetraene macrodiolides during the intramolecular cyclocondensation of ω-hydroxyalkadienoic acids with a (*Z*,*Z*)-1,5-diene fragment in the structure [41]. In order to expand the range of such unsaturated macrodiolides, we synthesized previously undescribed macrocyclic compounds based on α,ω-alka-n*Z*,(n+4)*Z*-dienediols **4a**–**d** and α,ω-alka-n*Z*,(n+4)*Z*-dienedioic acids **5a**–**d**. The synthesis of these compounds was accomplished through the application of established methodologies, drawing upon the fundamental reaction of catalytic homo-cyclomagnesiation of O-containing 1,2-dienes (Scheme 1) [37,38].

**Scheme 2:**
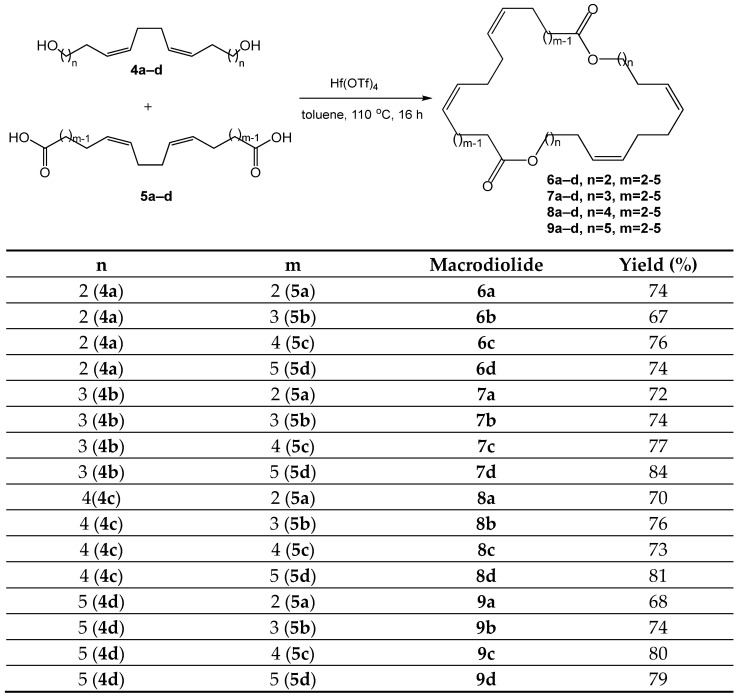
Synthesis of tetraenoic macrodiolides **6**–**9a**–**d**.

The target tetraene macrodiolides were synthesized via an intermolecular macrolactonization reaction of dienediols **4a**–**d** with dienedioic acids **5a**–**d**, in the presence of catalytic amounts of Hf(OTf)_4_ (Scheme 2) [42]. This one-pot preparative catalytic cyclocondensation method, developed by Collins et al. [43,44], has been demonstrated to be highly efficient in the synthesis of various macrodiolides and has been thoroughly established in the synthesis of diene macrolactones [37,38].

A preliminary investigation into the optimal reaction conditions was conducted, using the example of the interaction between 1,12-dodeca-4*Z*,8*Z*-dienediol **4a** and 1,12-dodeca-4*Z*,8*Z*-dienedioic acid **5a**. This investigation involved a series of experiments in which the amount of catalyst, the concentration of reacting substances in solution, and the reaction time were systematically varied.

It was ascertained that macrocyclization employing a 5 mol.% catalyst at the concentration of reactants [5 mM] results in the formation of a macrodiolide with the highest yield of 74%. Complete conversion is attained after 16 h of boiling in toluene. A subsequent increase in reaction time exerts a negligible effect on the yield of the target macrocycles. An increase in the amount of catalyst, up to 10 mol.%, has a negligible effect on the total yield and reaction time. However, a decrease in the amount of Hf(OTf)_4_ to 2.5 mol.% results in a decline in the total yield to 70%, accompanied by an increase in reaction time up to 30 h. When an increase in the concentration of the initial substrates is augmented to [10 mM], along with macrodiolides, the formation of cyclic and acyclic dimeric and oligomeric products is observed, the proportion of which can reach 50%. A decrease in the concentration of reactants to [3 mM] results in a slight decrease in the yield of the target reaction products to 68%, and complete conversion is achieved when the reaction is carried out for 40 h. Based on the experimental results obtained, the intermolecular cyclocondensation reactions between α,ω-alka-n*Z*,(n+4)*Z*-dienediols **4a**–**d** and α,ω-alka-n*Z*,(n+4)*Z*-dienedioic acid **5a**–**d** were carried out in toluene at boiling for 16–20 h using 5 mol.% catalyst and the concentration of initial reactants [5 mM]. It was observed that there was no discernible pattern in the reaction yields with respect to the carbon chain length of the reacting diol or dioic acid, which varied within the range of 67–84%.

### 2.2. Biological Studies

**Figure 2:**
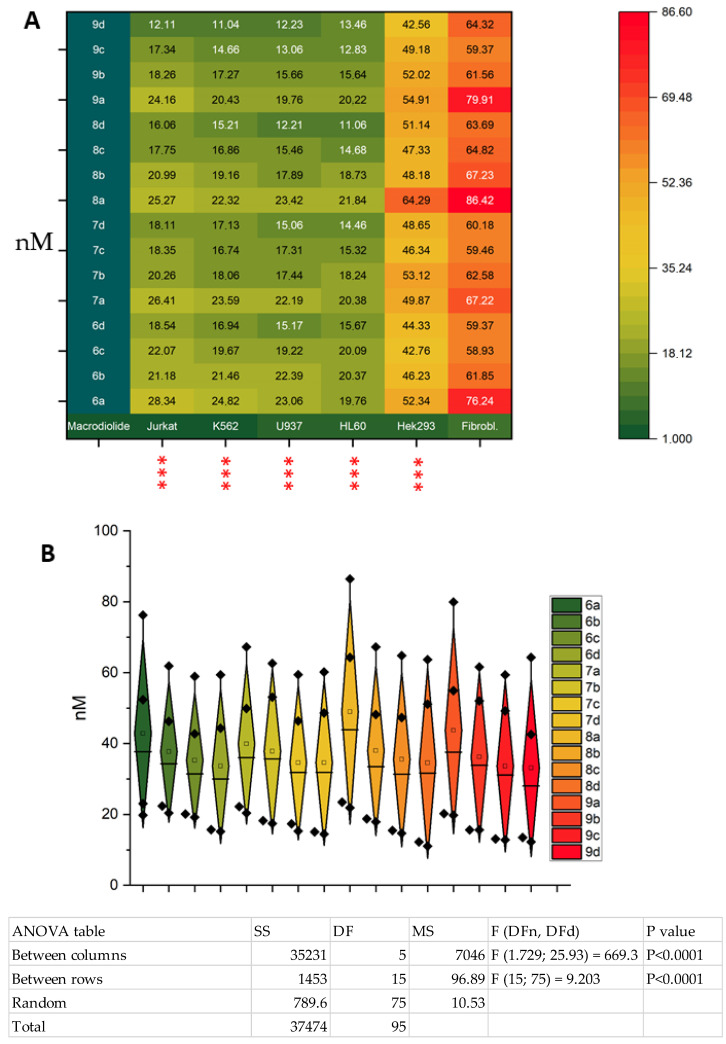
The cytotoxic activity (CC_50_) of the synthesized tetraene macrodiolides was evaluated in vitro on cell cultures (Jurkat, K562, U937, HL60, HEK293, and fibroblasts). (**A**) Heat map of the values representing the level of cell toxicity. (**B**) Histogram of standard deviation values. The values presented in the figure correspond to the median for all cell lines, with individual values for each cell line provided in the heatmap. CC_50_ was determined for all synthesized compounds. The incubation period was 24 h. The presence of three asterisks *** (*p* < 0.001) indicates the significance of the observed differences between the CC_50_ values obtained in the samples treated with the synthesized compounds and the CC_50_ values in fibroblast and the conditioned control cell line HEK293 (comparison was performed by one-way ANOVA). All experiments were performed in triplicate.

The investigation focused on the assessment of the synthesized compounds’ toxicities, utilizing a panel of six diverse cell lines. The selection of cells was based on the specific features of their metabolism, namely, Jurkat, K562, U937, and HL60 cells are suspension cultures of oncohematologic origin, and HEK293 is a conditionally normal line transformed by adenovirus and often used as control cells along with fibroblasts. The normal fibroblast line (Wi-38) utilized in the experiment was diploid and was obtained from the lung tissue of an aborted female fetus of the Caucasian race. The total cytotoxic activity of 16 compounds was studied using flow cytometry. The analysis of the obtained data on the cytotoxic activity of the synthesized compounds is presented in Table 1 and Figure 2.

The selectivity index was calculated by employing the following formula: CC_50_ for conditional normal cell line fibroblasts/CC_50_ for cancer cells. The following table illustrates the values of CC50 ± SD. All experiments were performed in triplicate.

- The following significant conclusions can be drawn from the analysis of the obtained data:
- The cytotoxic effect on the studied tumor lines increases in the series: Jurkat < K562 < U937 ≤ HL-60.
- A well-defined tendency of increasing toxicity of macrocycles with an increasing number of methylene links in dioic acid **5**, at a fixed number of carbon atoms in diol **4**, in each example for **4a**–**4d** is evident; the mentioned pattern is also observed for both the conditionally normal cell line Hek293 and the normal fibroblasts.
- The maximum cytotoxic effect on the Jurkat and K562 tumor lines was observed for compound **9d** (CC_50_ = 11.04 and 12.11), while for the U937 cell line, compounds **9d** and **8d** exhibited approximately equal maximum toxicity (CC_50_ = 12.21 and 12.23). For the HL60 cells, the maximum effect was observed for macrocycles **9c** and **9d** (CC_50_ = 12.83 and 13.46).

The evaluation of compound selectivity is recognized as a pivotal aspect in the realm of drug development. This critical parameter is meticulously monitored in numerous projects aimed at developing compounds of pharmacological significance, with the objective of minimizing the potential toxicity of these compounds. The selectivity index is a quantitative metric that quantifies the difference in toxicity of a compound to cancer cells and normal control cells. The selectivity of action exhibited by the synthesized compounds suggests the compounds’ potential to serve as drug candidates. The selectivity index demonstrates significant variability, ranging from approximately 2.5 to 5.3 for the Jurkat cells and from 3.0 to 5.8 for the other cell lines. A comparison of the selectivity indices for HEK293 and the fibroblasts revealed no statistically significant differences (see Table 1 and Figure 2).

It was observed that the new tetraene macrodiolides exhibited a higher level of cytotoxicity in comparison with the diene derivatives of macrocyclic compounds that were previously synthesized [37,38]. In general, only three diene macrodiolides exhibited cytotoxic activity comparable to tetraenes (CC_50_ = 0.01–0.03 µM) on individual cell cultures (Jurkat, U937). Therefore, the most active diene macrodiolide obtained from 1,18-octadeca-7*Z*,11*Z*-dienediol and pimelic acid exhibited comparable toxicity to tetraene macrocycles, selectively targeting the Jurkat and U937 cell lines (CC_50_ = 0.01–0.02 µM). However, its activity on the other investigated cell cultures, i.e., K562, HEK293, and HeLa, was lower (CC_50_ = 0.06–0.15 µM). The other diene macrodiolides synthesized based on α,ω-alka-n*Z*-(n+4)*Z*-dienediols demonstrated cytotoxic activity on the studied cell lines (Jurkat, K562, Hek293, HeLa, U937) within the range of CC_50_ = 0.03–0.42 µM [37]. The cytotoxic activity of all the diene macrodiolides derived from α,ω-alka-n*Z*,(n+4)*Z*-dienedioic acid ranged between CC_50_ = 0.03–0.93 µM on Jurkat, K562, U937, HEK293, and HeLa cell cultures [38].

**Figure 3:**
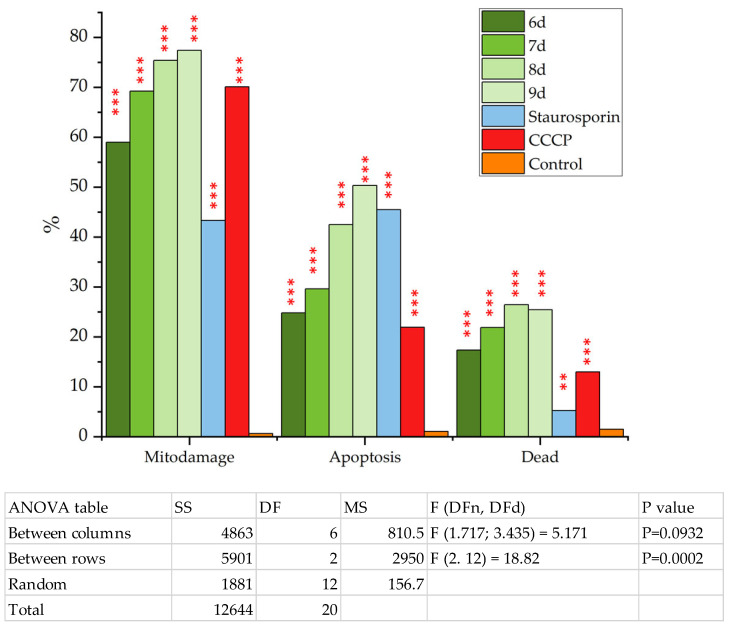
Detection changes in mitochondrial membrane potential (ΔΨ) and coupled early and late apoptosis in Jurkat cells treated with compounds **9d**, **8d**, **7d**, and **6d**. Staurosporine and carbonyl cyanide-m-chlorophenylhydrazone were utilized as control compounds. The addition of all the tested compounds was executed at the CC_50_ concentration (refer to the provided table for the cytotoxicities of the synthesized compounds). The staining of cells was conducted using MitoSense Red, Annexin V-CF488A, and 7-AAD. The incubation period lasted for a duration of four hours. The presence of three *** (*p* < 0.001) and two asterisks ** (*p* < 0.005) indicates the significance of the observed differences between the values obtained in the control sample and the samples treated with the synthesized substances (comparison was performed by one-way ANOVA). All experiments were performed in triplicate.

The present study investigated the changes in mitochondrial membrane potential (ΔΨ) in Jurkat cells following treatment with the compounds that demonstrated the greatest degree of toxicity: **9d**, **8d**, **7d**, and **6d**. To this end, MitoSense Red, a fluorescent dye that accumulates predominantly in mitochondria and responds to changes in membrane potential, was utilized. Our earlier studies demonstrated that macrodiolides exhibit divergent biological effects on mitochondria [38,39,40]. The analysis method employed is outlined as follows: live full-grown cells exhibit a high level of MitoSense Red fluorescence, while cells experiencing dissipation of mitochondrial membrane potential demonstrate a significantly lower or absent MitoSense Red fluorescence. Two dyes commonly employed for detecting apoptosis in cells are annexin V and 7AAD. Control cells exhibit no fluorescence, while apoptotic cells manifest positive green fluorescence due to externalization of phosphotidylserine. It is important to note that 7-aminoactinomycin (7-AAD) does not penetrate living cells; rather, it serves as a marker for changes in cell membrane permeability during the late stages of apoptosis and in dead cells. Therefore, the dissipation of mitochondrial membrane potential and the associated apoptosis process in Jurkat tumor cells were analyzed in response to exposure to synthesized macrodiolides. MitoSense Red, Annexin V-CF488A, and 7-AAD were used as dyes for this analysis (see Figure 3).

A significant increase in the content of the Jurkat tumor cells with a reduced level of mitochondrial membrane potential dissipation (Δψ) was found in the samples treated with compounds **9d** (77.42%), **8d** (75.42%), **7d** (69.26%), and **6d** (58.99%) (Figure 3). Staurosporine and CCCP (carbonyl cyanide-m-chlorophenylhydrazone) were utilized as control compounds. Compound **9d** exhibited the most pronounced effect on mitochondrial potential, which was comparable to the effect of the well-known dissipator compound CCCP (Figure 3 and Table S1 in Supplementary Materials). The percentage of cells in apoptosis in the samples treated with compound **9d** was 50.34% (Figure 3 and Table S1 in Supplementary Materials). The obtained data indicate the initiation of apoptosis through the internal (mitochondrial) pathway, and compound **9d** was the most active dissipator of mitochondrial potential because of a pronounced decrease in oxidation and phosphorylation processes in mitochondria of the Jurkat cells.

A reliable confirmation of apoptosis activation via the mitochondrial pathway is the detection of cytochrome c release into the cytoplasm. The role of cytochrome c as an inducer of apoptosis was confirmed in experiments where the addition of deoxyadenosine triphosphate (dATP) to cytosolic extracts caused an increase in caspase activity. The absence of cytochrome c resulted in a lack of development of this activity [45]. Subsequent studies demonstrated the pivotal role of cytochrome c in this process, establishing a direct connection between its activation and the induction of apoptosis [46]. Cytochrome c plays a pivotal role in the initiation of both the intrinsic and extrinsic apoptosis pathways. The initiation of internal apoptosis is triggered by DNA damage, metabolic stress, or the presence of proteins with a disturbed structure. The pivotal step in this process is the permeabilization of the mitochondrial outer membrane. Upon activation of the processes of cell damage and apoptosis development, cytochrome leaves the mitochondrial intermembrane space into the cytoplasm and binds to the apoptotic protease activation factor (Apaf-1) [47]. Consequently, the release of cytochrome C into the cytoplasm instigates the formation of the apoptosome.

**Figure 4:**
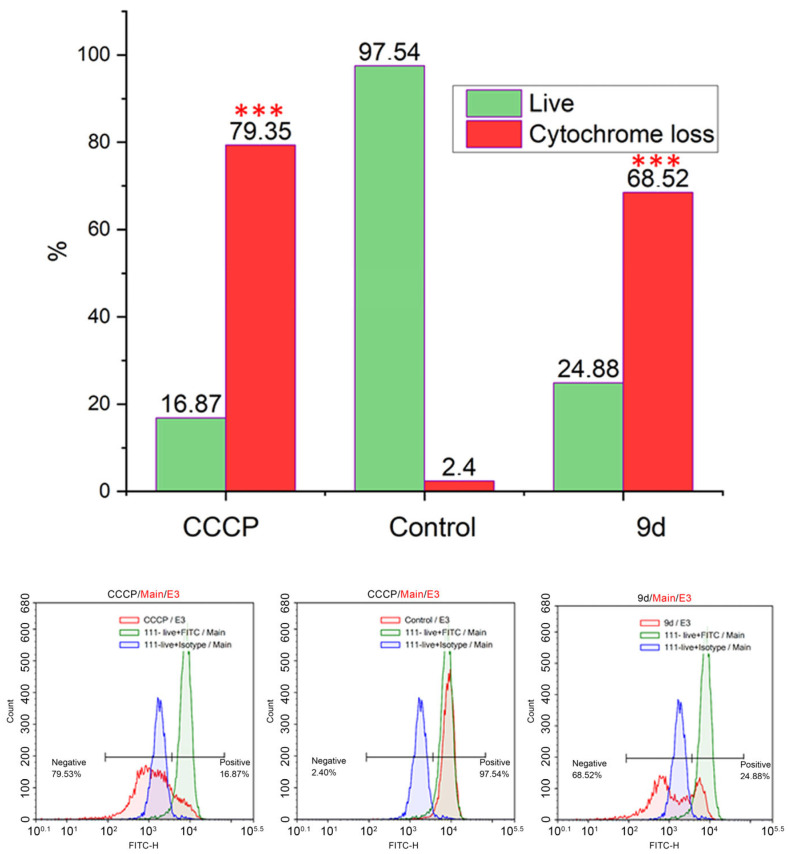
Detection of cytochrome c in Jurkat cells stained with a FlowCellect Cytochrome c Kit. The control compound, carbonyl cyanide m-chlorophenylhydrazone (CCCP), was administered at a concentration equal to its CC50 value. Compound **9d** was added to the sample cells at a CC50 concentration (see the table of cell viability). The cells were incubated for 6 h. We compared the numerical values to determine the reliability of the differences observed between the control sample and the samples treated with the synthesized substances. The presence of three asterisks *** (*p* < 0.001) indicates the significance of the observed differences between the values obtained in the control sample and the samples treated with the synthesized substances. We used a statistical *t*-test to perform this comparison. All experiments were performed in triplicate.

An investigation was conducted into the presence of cytochrome C in the cytoplasm of the cells treated with compound **9d**. The universal dissipator carbonyl cyanide-m-chlorophenylhydrazone (CCCP) was utilized as a positive control. The results of this investigation are illustrated in Figure 4, which presents the cytometric detection of cytochrome C in the cytoplasm of the treated cells.

As demonstrated in Figure 4, compound leader **9d** induces cytochrome release from mitochondria and exhibits a significant difference compared to the control sample. It is noteworthy that compound **9d** exhibits a comparable effect to the well-studied mitochondrial respiratory chain uncoupler, carbonyl cyanide m-chlorophenylhydrazone. Treatment with compound **9d** resulted in 68.22% of the cells releasing cytochrome c into the cytoplasm, while treatment with CCCP achieved 79.35% cytochrome c release. A comparison of **9d** with m-chlorophenylhydrazone carbonyl cyanide reveals that the former causes slightly greater dissipation of the mitochondrial membrane. Consequently, the synthesized tetraene macrodiolides exhibit compatibility with the action of CCCP on the mitochondrial membrane. These compounds, which exhibit a pronounced action as dissociators of mitochondrial potential, may have utility as antitumor agents.

## 3. Materials and Methods

### 3.1. Chemistry

NMR spectra were recorded in CDCl_3_ on Bruker Ascend-500 ((500 MHz (^1^H), 126 MHz (^13^C)) instruments. Mass spectra were obtained on an UltraFlex III TOF/TOF (Bruker Daltonik GmbH, Bremen, Germany) operating in linear (TOF) and reflection (TOF/TOF) positive and negative ion modes. The synthesized products were isolated using a SepaBean machine (Santai Science Inc., Montréal, QC, Canada) flash chromatography instrument (flow rate of 1–200 mL min^−1^, maximum pressure 13.8 bar, DAD UV (200–400 nm) detector or DAD UV (200–400 nm) + Vis (400–800 nm) detector); Standard Series flash columns (12 g) packed with UltraPure irregular silica gel (40–63 µm, 60 Å); or high-efficiency irregular silica gel (25–40 µm, 60 Å) using hexane–EtOAc mixtures of increasing polarity. The isolated yields reflect the mass obtained following flash column silica gel chromatography. All solvents were dried (diethyl ether, toluene over Na, dioxane over NaOH, methanol over Mg, chloroform over P_2_O_5_) and freshly distilled before use. All reactions were carried out under a dry argon atmosphere. Commercially available alkynols (pent-4-yn-1-ol, hex-5-yn-1-ol, hept-6-yn-1-ol, oct-7-yn-1-ol) (TCI), titanocene dichloride (Cp_2_TiCl_2_), and hafnium (IV) trifluoromethansulfonate (Hf(OTf)_4_) (Aldrich) were used. The synthesis and analytical data of α,ω-alka-n*Z*,(n+4)*Z*-dienediols (1,12-dodeca-4Z,8Z-dienediol (**4a**), 1,14-tetradeca-5Z,9Z-dienediol (**4b**), 1,16-hexadeca-6Z,10Z-dienediol (**4c**), 1,18-octadeca-7Z,11Z-dienediol (**4d**)) were described earlier in the articles [37,41]. The details of the characterization of all the newly synthesized compounds are available in the Supplementary Material.

### 3.2. Synthetic Procedures

#### 3.2.1. The General Procedure for the Synthesis of α,ω-Alka-n*Z*,(n+4)*Z*-Dienedioic Acid **5a**–**d**

The oxidation procedure used by Corey et al. [48] was followed, with some modifications. To a stirred solution of pyridinium dichromate (2.63 g, 7.0 mmol) in anhydrous DMF (9 mL), a solution of α,ω-alka-n*Z*,(n+4)*Z*-dienediol 4a-d (1.0 mmol) in DMF (1 mL) was added. The reaction mixture was stirred at room temperature (20–22 °C) for 16 h, washed with water, and extracted with diethyl ether (3 × 20 mL). The combined organic layers were dried with MgSO_4_ and concentrated in vacuo. For products 5a-d, flash chromatography (40% EtOAc in hexanes) was performed to afford the desired product as a yellowish oil. The chemical experimental data synthesized for α,ω-alka-n*Z*,(n+4)*Z*-dienedioic acids were published earlier [38].

#### 3.2.2. The General Procedure for the Synthesis of Tetraene Macrodiolides **6a**–**9d**

The synthesis of compounds **6a**–**9d** was carried out similarly to the known procedure [44]. α,ω-alka-n*Z*,(n+4)*Z*-dienediol (0.2 mmol, 1.0 equiv.) and α,ω-alka-n*Z*,(n+4)*Z*-dienedioic acid (0.2 mmol, 1.0 equiv.) were dissolved in toluene (40 mL, 5 mM). Then, Hf(OTf)_4_ (0.01 mmol, 0.05 equiv.) was added to the solution, and the reaction mixture was heated to 110 °C and stirred at this temperature for 16–20 h. Then, the reaction mixture was concentrated under reduced pressure and purified by flash chromatography (5% EtOAc in hexanes). The analytical data of the synthesized macrodiolides are given in the Supplementary Material.

### 3.3. Biological Studies

#### 3.3.1. Cell Culturing

Human cancer cell lines, including Jurkat, K562, U937, HL60, and fibroblasts (Wi-38), were obtained from the European Authentic Cell Culture Collection and further cultured according to established standard protocols using sterile methods. The cells were cultivated in RPMI 1640 (for Jurkat and K562) and DMEM (for Wi-38 and HEK293) media (Gibco, Billings, MT, USA), with the addition of 4 μM glutamine, 10% FBS (Sigma, Kanagawa, Japan), and 100 U/mL penicillin–streptomycin (Sigma). All cell types were cultivated in a humidified atmosphere containing 5% CO₂ at a temperature of 37 °C. Subculturing was performed at two- to three-day intervals. The cells were subsequently seeded into 24-well plates at a density of 5 × 10^4^ cells per well and incubated overnight. Subsequent subculturing was then performed at two-day intervals, with a seeding density of 1 × 10^5^ cells per 24-well plate, using RPMI medium with 10% FBS [49].

#### 3.3.2. Preparation of Effluent and Test Compound Solutions for Biological Testing

The dissolution of the compounds tested was initially performed in dimethyl sulfoxide (DMSO) with an initial solution of 100 mM in 10% DMSO. Subsequently, the solution was diluted in a complete culture medium, namely, Dulbecco’s medium, modified Eagle’s medium (DMEM), or Roswell Park Memorial Institute medium (RPMI). Substances were added at concentrations of 10, 1, 100, 10, 1, and 0.1 μM on the day after seeding and incubated for 24 h. Following this, the cells were washed with a phosphate-buffered saline (PBS) solution and stained with 7AAD dye, as per the manufacturer’s protocol for flow cytometry. The CC50 value, which characterizes the parameters of toxicity (i.e., the concentration of the compound required for 50% inhibition of cell viability in vitro), was calculated. logC versus % inhibition was plotted, and statistical data processing was performed using Excel and GraphPad Prism v.8.0.2 (San Diego, CA, USA, 2019). The data obtained from three independent experiments were expressed as the mean of three measurements for each concentration, with the standard deviation indicated as well. These values were then relative to the control values (0.1% DMSO), which were taken as 100%.

#### 3.3.3. Cytotoxicity Assay

7-AAD (7-aminoactinomycin D) dye (eBioscience™, San Diego, CA, USA) was used to assess cell viability. Following incubation with the test compounds, the cells were harvested, washed with a phosphate–salt buffer (PBS), and subjected to centrifugation at 400× *g* for five minutes. The cell sediment was resuspended in 200 μL of staining buffer for flow cytometry (PBS without calcium and magnesium, 2.5% FBS) and stained with a 1 mM 7-AAD dye solution for 15 min in the dark at room temperature. Subsequently, all samples, including experimental and control groups, were analyzed using a BD FACSAria™ III Cell Sorter flow cytometer (Milpitas, CA, USA).

#### 3.3.4. Mitodamage Assay

A flow cytometry kit (FlowCellect™ MitoDamage Kit from Millipore, Hayward, CA USA) was utilized, as it facilitates a multiparametric evaluation of three markers of cell health. These markers include changes in mitochondrial potential (early apoptosis and cell stress), phosphatidylserine expression on the cell surface (late apoptosis), and membrane permeability (cell death). The method of the simultaneous detection of mitochondrial damage was performed according to the well-known technique [38].

#### 3.3.5. Cytochrome C Assay

The quantification of cytochrome c release from the mitochondria in apoptotic cells was used to detect the mitochondrial pathway of apoptosis in the cells by flow cytometry. This study employed a direct labeling method that utilized an anti-cytochrome cFITC antibody, an anti-IgG1-FITC isotype control, and optimized fixation, permeabilization, and blocking buffers to detect cytochrome c by flow cytometry (Luminex^®^, Austin, TX, USA). The results demonstrated that cytochrome c fluorescence levels were higher in living cells and lower in apoptotic cells, indicating the release of cytochrome c from the mitochondria into the cytoplasm. The Jurkat cells were exposed to test substances at concentrations corresponding to their 24-h CC50 in these cells for a period of four hours. Thereafter, analysis by flow cytometry (NovoCyte Flow Cytometry™ Agilent, San Diego, CA, USA) was conducted. The acquired data were processed using NovoExpress^®^ software (version 1.6.2) (ACEA (Agilent, Santa Clara, CA, USA)) [50].

#### 3.3.6. Statistics

The mean values and standard errors were calculated for each data set. Subsequently, group comparisons and statistical analysis were performed using one-way ANOVA, followed by Fisher’s multiple comparisons test. The analysis and graphical representation of the data were conducted using GraphPad Prism v6 (GraphPad Software, San Diego, CA, USA) and Origin Pro 2024 (OriginPro 2024 SR1 10.1.0.178, OriginLab Corporation, Northampton, MA, USA). CC50 values and the selectivity index were calculated using GraphPad Prism v6 software (GraphPad Software, San Diego, CA, USA).

## 4. Conclusions

The directed synthesis of isomerically pure tetraene macrodiolides containing two pharmacophore *cis*,*cis*-1,5-diene fragments in their structure was carried out for the first time. The in vitro cytotoxic potential of the synthesized macrodiolides was evaluated using Jurkat, K562, U937, and HL-60 cancer cell lines. The selectivity index of each synthesized compound was also determined. This work revealed that the new tetraene macrodiolides exhibited high levels of cytotoxic activity in vitro compared to previously published diene derivatives. Furthermore, evidence was presented demonstrating that tetraene macrodiolides induce mitochondrial-type apoptosis due to the release of cytochrome C into the cytosol. It is hypothesized that macrocyclic compounds, because of their lipophilicity, are capable of transporting protons (protonophores) or cations (ionophores) across the inner membrane, thereby bypassing a specialized channel in F0 located at the interface between the subunits a and c of ATP synthase. Consequently, the H+ gradient may undergo a decrease, the ADP content may increase, and the rate of oxidation and O_2_ uptake may increase, but energy is dissipated as heat, and the P/O ratio may decrease. Dinitrophenol, valinomycin, gramicidin, dicoumarol, bilirubin, uric acid, thyroxine, and long-chain fatty acids have been observed to act in a similar manner. The process of distinguishing between oxidation and phosphorylation has been shown to increase the production of heat. This phenomenon is exemplified by the presence of brown and beige fat tissues, which are characterized by their high concentration of mitochondria and the presence of the thermogenin protein. The function of the thermogenin protein is to facilitate the transport of fatty acids into the matrix. These compounds demonstrate considerable promise for utilization in medical applications. These agents may be utilized to address a wide range of processes, including, but not limited to, obesity and tumor growth. The significance of these compounds lies in their role in regulating energy balance processes within mitochondria, thereby establishing them as essential constituents for the development of future pharmaceutical substances.

## Figures and Tables

**Table 1:** In vitro cytotoxic activities of synthesized tetraene macrodiolides **6**–**9** measured on cell cultures (Jurkat, K562, U937, HL60, Hek293, and normal fibroblasts) (nM).

Macrodiolide	Jurkat (CC50, nM)	SI *	K562 (CC50, nM)	SI	U937 (CC50, nM)	SI	HL60 (CC50, nM)	SI	Hek293 (CC50, nM)	SI	Fibrobl. (CC50, nM)
**6a**	28.34 ± 2.65	2.6	24.82 ± 2.37	3.0	23.06 ± 2.11	3.3	19.76 ± 1.81	3.8	52.34 ± 5.17	1.4	76.24 ± 7.62
**6b**	21.18 ± 1.97	2.9	21.46 ± 2.01	2.8	22.39 ± 2.24	2.7	20.37 ± 2.10	3.0	46.23 ± 3.75	1.3	61.85 ± 6.27
**6c**	22.07 ± 2.14	2.6	19.67 ± 1.63	2.9	19.22 ± 1.87	3.0	20.09 ± 1.95	2.9	42.76 ± 4.39	1.3	58.93 ± 5.81
**6d**	18.54 ± 1.78	3.2	16.94 ± 1.88	3.5	15.17 ± 1.56	3.9	15.67 ± 1.43	3.7	44.33 ± 4.21	1.3	59.37 ± 5.46
**7a**	26.41 ± 2.46	2.5	23.59 ± 2.14	2.8	22.19 ± 2.34	3.0	20.38 ± 2.07	3.2	49.87 ± 4.83	1.3	67.22 ± 6.39
**7b**	20.26 ± 2.04	3.0	18.06 ± 1.83	3.4	17.44 ± 1.71	3.5	18.24 ± 1.76	3.4	53.12 ± 4.96	1.1	62.58 ± 5.94
**7c**	18.35 ± 1.86	3.2	16.74 ± 1.59	3.5	17.31 ± 1.62	3.4	15.32 ± 1.48	3.8	46.34 ± 3.99	1.2	59.46 ± 5.37
**7d**	18.11 ± 1.91	3.3	17.13 ± 1.84	3.5	15.06 ± 1.43	3.9	14.46 ± 1.57	4.1	48.65 ± 4.82	1.2	60.18 ± 5.21
**8a**	25.27 ± 2.63	3.4	22.32 ± 2.16	3.8	23.42 ± 2.27	3.6	21.84 ± 1.96	3.9	64.29 ± 5.37	1.3	86.42 ± 7.11
**8b**	20.99 ± 2.34	3.2	19.16 ± 1.79	3.5	17.89 ± 1.83	3.7	18.73 ± 1.71	3.5	48.18 ± 4.34	1.3	67.23 ± 6.24
**8c**	17.75 ± 1.73	3.6	16.86 ± 1.62	3.8	15.46 ± 1.49	4.1	14.68 ± 1.42	4.4	47.33 ± 4.22	1.3	64.82 ± 6.35
**8d**	16.06 ± 1.58	3.9	15.21 ± 1.46	4.1	12.21 ± 1.37	5.2	11.06 ± 1.39	5.7	51.14 ± 5.60	1.2	63.69 ± 6.07
**9a**	24.16 ± 2.39	3.3	20.43 ± 1.89	3.9	19.76 ± 1.97	4.0	20.22 ± 2.17	3.9	54.91 ± 5.37	1.4	79.91 ± 6.90
**9b**	18.26 ± 2.04	3.3	17.27 ± 1.64	3.5	15.66 ± 1.53	3.9	15.64 ± 1.49	3.9	52.02 ± 5.43	1.1	61.56 ± 5.92
**9c**	17.34 ± 1.59	3.4	14.66 ± 1.49	4.0	13.06 ± 1.31	4.5	12.83 ± 1.37	4.6	49.18 ± 4.77	1.2	59.37 ± 5.29
**9d**	12.11 ± 1.77	5.3	11.04 ± 1.25	5.8	12.23 ± 1.41	5.2	13.46 ± 1.21	4.7	42.56 ± 4.31	1.5	64.32 ± 5.81

* The red palette corresponds to a decrease in SI values, while the transition to green indicates an increase in values.

## Data Availability

The data are available upon request.

## Supplementary Materials

The following supporting information can be downloaded at https://www.mdpi.com/article/10.3390/ijms26115139/s1.
