# Supplementary material for: Novel Tetraene Macrodiolides Are Effective Inducers of Mitochondrial Apoptosis in Jurkat Cells
Source: Int J Mol Sci. 2025 May 27;26(11):5139. doi: 10.3390/ijms26115139 (PMC12155470; doi:10.3390/ijms26115139)
Supplement: Supplementary file 1 [file ijms-26-05139-s001.zip › ijms-3606439-supplementary.pdf]

Novel Tetraene Macrodiolides Are Effective Inducers of Mitochondrial Apoptosis in Jurkat Cells

Ilgis I. Islamov [1], Lilya U. Dzhemileva [2], Ilgam V. Gaisin [1], Alexey A. Makarov [1], Usein M. Dzhemilev [2], and Vladimir A. D'yakonov [2]

[1] *Institute of Petrochemistry and Catalysis, Russian Academy of Sciences, 141 Prospekt Oktyabrya, Ufa 450075, Russian Federation*

[2] *N. D. Zelinsky Institute of Organic Chemistry, Russian Academy of Sciences, Leninsky Prospekt, 47, Moscow 119991, Russian Federation*

E-mail: iislamovi@gmail.com,

E-mail: dzhemilev@mail.ru

Table of Contents

Chemical Experimental Data of compounds 6a-9d……………………………………………..S3

^{1}H NMR and ^{13}C NMR spectra of compounds 6a-9d………………………..……………......S5

Table S1: Detection of changes in mitochondrial membrane potential (ΔΨ) and the associated early and late stages of apoptosis in Jurkat cells treated with compounds 6-9d, CCCP, and staurosporine…………….…………………………………………………………………………S19

Chemical Experimental Data of compounds 6a-9d

(5Z,9Z,18Z,22Z)-1,14-dioxacyclohexacosa-5,9,18,22-tetraene-2,13-dione (6a). Yellow oil; yield 74%. ^{1}H NMR (500 MHz, $CDCl_3$): δ = 5.55 – 5.36 (m, 8H), 4.10 (t, *J* = 6.2 Hz, 4H), 2.41– 2.31 (m, 8H), 2.17 – 2.03 (m, 12H), 1.73 – 1.66 (m, 4H). ^{13}C NMR (126 MHz, $CDCl_3$): δ = 173.1, 130.6, 130.1, 128.9, 128.1, 63.7, 34.6, 28.5, 27.4, 27.3, 23.6, 23.0. ESI-MS: calcd. for $C_{24}H_{36}O_4 + H^+$ $[M + H]^+$ 389,2686; found 389,2653.

(5Z,9Z,19Z,23Z)-1,14-dioxacyclooctacosa-5,9,19,23-tetraene-15,28-dione (6b). Yellow oil; yield 67%. 1H NMR (500 MHz, CDCl3): δ = 5.45–5.34 (m, 8H), 4.09 (t, J = 6.2 Hz, 4H), 2.33 (t, J = 7.3 Hz, 4H), 2.23–1.82 (m, 16H), 1.82–1.63 (m, 8H). 13C NMR (126 MHz, CDCl3): δ = 173.7, 130.3, 130.2, 129.0, 128.8, 63.6, 33.6, 28.6, 27.4, 27.3, 26.5, 24.8, 23.6. ESI-MS: calcd. for C26H40O4 + H+ [M + H]+ 417,2999; found 417.2985.

(5Z,9Z,20Z,24Z)-1,14-dioxacyclotriaconta-5,9,20,24-tetraene-15,30-dione (6c). Yellow oil; yield 76%. ^{1}H NMR (500 MHz, $CDCl_3$): δ = 5.51 – 5.31 (m, 8H), 4.09 (t, *J* = 6.1 Hz, 4H), 2.33 (td, *J* = 7.5, 4.2 Hz, 4H), 2.16 – 1.96 (m, 16H), 1.78 – 1.54 (m, 12H). ^{13}C NMR (126 MHz, $CDCl_3$): δ = 173.7, 130.2, 129.7, 129.6, 128.8, 63.6, 34.3, 29.1, 28.6, 27.5, 27.3, 26.7, 24.6, 23.7. ESI-MS: calcd. for $C_{28}H_{44}O_4$ + Na^+ $[M + Na]^+$ 467,3132; found 467.3136.

(5Z,9Z,21Z,25Z)-1,14-dioxacyclodotriaconta-5,9,21,25-tetraene-15,32-dione (6d). Yellow oil; yield 74%. ^{1}H NMR (500 MHz, $CDCl_3$): δ = 5.47 – 5.35 (m, 8H), 4.10 (t, *J* = 6.0 Hz, 4H), 2.32 (t, *J* = 7.2 Hz, 4H), 2.16 – 1.93 (m, 16H), 1.73 – 1.60 (m, 8H), 1.43 – 1.34 (m, 8H). ^{13}C NMR (126 MHz, $CDCl_3$): δ = 173.9, 130.2, 129.9, 129.4, 128.8, 63.6, 34.4, 29.3, 28.9, 28.6, 27.5, 27.3, 26.9, 24.9, 23.7. ESI-MS: calcd. for $C_{30}H_{48}O_4 + Na^+$ $[M + Na]^+$ 495,3445; found 495,3441.

(5Z,9Z,19Z,23Z)-1,14-dioxacyclooctacosa-5,9,19,23-tetraene-2,13-dione (7a). Yellow oil; yield 72%. ^{1}H NMR (500 MHz, $CDCl_3$): δ = 5.47 – 5.36 (m, 8H), 4.10 (t, *J* = 6.6 Hz, 4H), 2.41– 2.33 (m, 8H), 2.15 – 2.01(m, 12H), 1.68 – 1.61 (m, 4H), 1.47 – 1.42 (m, 4H). ^{13}C NMR (126 MHz, $CDCl_3$): δ = 173.2, 130.6, 129.7, 129.6, 128.0, 64.3, 34.6, 28.2, 27.4, 27.3, 26.7, 26.0, 23.0. ESI-MS: calcd. for $C_{26}H_{40}O_4$ + H^+ $[M + H]^+$ 417,2999; found 417,3000.

(6Z,10Z,21Z,25Z)-1,16-dioxacyclotriaconta-6,10,21,25-tetraene-2,15-dione (7b). Yellow oil; yield 74%. ^{1}H NMR (500 MHz, $CDCl_3$): δ = 5.45–5.33 (m, 8H), 4.09 (t, *J* = 6.6 Hz, 4H), 2.32 (t, *J* = 7.4 Hz, 4H), 2.12–1.94 (m, 16H), 1.74–1.57 (m, 8H), 1.47–1.38 (m, 4H). ^{13}C NMR (126 MHz, $CDCl_3$): δ = 173.7, 130.2, 129.7, 129.6, 129.0, 64.2, 33.6, 28.2, 27.4, 27.3, 26.7, 26.5, 26.1, 24.8. ESI-MS: calcd. for $C_{28}H_{44}O_4 + H^+$ $[M + H]^+$ 445.3312; found 445.3321.

(6Z,10Z,22Z,26Z)-1,16-dioxacyclodotriaconta-6,10,22,26-tetraene-17,32-dione (7c). Yellow oil; yield 77%. ^{1}H NMR (500 MHz, $CDCl_3$): δ = 5.44 – 5.35 (m, 8H), 4.09 (t, *J* = 6.6 Hz, 4H), 2.32 (t, *J* = 7.5 Hz, 4H), 2.12 – 2.00 (m, 16H), 1.69 – 1.63 (m, 8H), 1.47 – 1.38 (m, 8H). ^{13}C NMR (126 MHz, $CDCl_3$): δ = 173.8, 129.7, 129.7, 129.6, 129.6, 64.2, 34.3, 29.1, 28.2, 27.5, 27.4, 26.8, 26.7, 26.1, 24.7. ESI-MS: calcd. for $C_{30}H_{48}O_4 + Na^+$ $[M + Na]^+$ 495,3445; found 495,3452.

(6Z,10Z,23Z,27Z)-1,16-dioxacyclotetratriaconta-6,10,23,27-tetraene-17,34-dione (7d). Yellow oil; yield 84%. ^{1}H NMR (500 MHz, $CDCl_3$): δ = 5.46 – 5.33 (m, 8H), 4.09 (t, *J* = 6.6 Hz, 4H), 2.32 (t, *J* = 7.4 Hz, 4H), 2.11 – 2.01 (m, 16H), 1.68 – 1.62 (m, 8H), 1.46 – 1.33 (m, 12H). ^{13}C NMR (126 MHz, $CDCl_3$): δ = 173.9, 130.0, 129.7, 129.6, 129.4, 64.2, 34.4, 29.3, 28.7, 28.2, 27.5, 27.4, 26.9, 26.7, 26.0, 24.9.ESI-MS: calcd. for $C_{32}H_{52}O_4 + Na^+$ $[M + Na]^+$ 523,3758; found 523,3693.

(5Z,9Z,20Z,24Z)-1,14-dioxacyclotriaconta-5,9,20,24-tetraene-2,13-dione (8a). Yellow oil; yield 70%. ^{1}H NMR (500 MHz, $CDCl_3$): δ = 5.48 – 5.34 (m, 8H), 4.09 (t, J = 6.5 Hz, 4H), 2.41 – 2.33 (m, 8H), 2.15 – 1.99 (m, 12H), 1.66 – 1.60 (m, 4H), 1.43 – 1.35 (m, 8H). ^{13}C NMR (126 MHz, $CDCl_3$): δ = 173.2, 130.5, 129.9, 129.5, 128.1, 64.4, 34.6, 29.2, 28.5, 27.5, 27.3, 26.9, 25.5, 23.0. ESI-MS: calcd. for $C_{28}H_{44}O_4$ + Na^+ $[M + Na]^+$ 467.3132; found 467.3146.

(6Z,10Z,22Z,26Z)-1,16-dioxacyclodotriaconta-6,10,22,26-tetraene-2,15-dione (8b). Yellow oil; yield 76%. ^{1}H NMR (500 MHz, $CDCl_3$): δ = 5.44–5.34 (m, 8H), 4.08 (t, J = 6.4 Hz, 4H), 2.32 (t, J = 7.4 Hz, 4H), 2.12–1.95 (m, 16H), 1.73–1.62 (m, 8H), 1.40–1.32 (m, 8H). ^{13}C NMR (126 MHz, $CDCl_3$): δ = 173.7, 130.2, 129.9, 129.5, 129.0, 64.3, 33.6, 29.2, 28.5, 27.5, 27.4, 26.9, 26.5, 25.6, 24.9. ESI-MS: calcd. for $C_{30}H_{48}O_4$ + H^+ $[M + H]^+$ 473.3625; found 473.3652.

(7Z,11Z,24Z,28Z)-1,18-dioxacyclotetratriaconta-7,11,24,28-tetraene-2,17-dione (8c). Yellow oil; yield 73%. ^{1}H NMR (500 MHz, $CDCl_3$): δ = 5.48 – 5.32 (m, 8H), 4.08 (t, J = 6.4 Hz, 4H), 2.32 (t, J = 7.4 Hz, 4H), 2.10 –2.01 (m, 16H), 1.72 –1.63 (m, 8H), 1.45 –1.31 (m, 12H). ^{13}C NMR (126 MHz, $CDCl_3$): δ = 173.8, 129.9, 129.7, 129.6, 129.5, 64.3, 34.3, 29.2, 29.1, 28.5, 27.5, 27.4, 27.0, 26.8, 25.6, 24.7. ESI-MS: calcd. for $C_{32}H_{52}O_4$ + Na^+ $[M + Na]^+$ 523.3758; found 523.3743.

(7Z,11Z,25Z,29Z)-1,18-dioxacyclohexatriaconta-7,11,25,29-tetraene-19,36-dione (8d). Yellow oil; yield 81%. ^{1}H NMR (500 MHz, $CDCl_3$): δ = 5.41 – 5.36 (m, 8H), 4.09 (t, J = 6.4 Hz, 4H), 2.31 (t, J = 7.4 Hz, 4H), 2.10 – 1.93 (m, 16H), 1.68 –1.57 (m, 8H), 1.45 –1.34 (m, 16H). ^{13}C NMR (126 MHz, $CDCl_3$): δ = 173.9, 130.0, 129.9, 129.5, 129.4, 64.3, 34.4, 29.3, 29.2, 28.7, 28.5, 27.5, 27.4, 27.0, 26.9, 25.6, 24.9. ESI-MS: calcd. for $C_{34}H_{56}O_4$ + Na^+ $[M + Na]^+$ 551.4071; found 551.3978.

(5Z,9Z,21Z,25Z)-1,14-dioxacyclodotriaconta-5,9,21,25-tetraene-2,13-dione (9a). Yellow oil; yield 68%. ^{1}H NMR (500 MHz, $CDCl_3$): δ =5.45 – 5.36 (m, 8H), 4.08 (t, J = 6.6 Hz, 4H), 2.40 – 2.30 (m, 8H), 2.13 – 2.03 (m, 12H), 1.65 – 1.57 (m, 4H), 1.44 – 1.27 (m, 12H). ^{13}C NMR (126 MHz, $CDCl_3$): δ = 173.2, 130.6, 130.1, 129.3, 128.0, 64.5, 34.6, 29.5, 28.7, 28.6, 27.5, 27.2, 26.9, 25.8, 22.9. ESI-MS: calcd. for $C_{30}H_{48}O_4$ + Na^+ $[M + Na]^+$ 495,3445; found 495,3435.

(6Z,10Z,23Z,27Z)-1,16-dioxacyclotetratriaconta-6,10,23,27-tetraene-2,15-dione (9b). Yellow oil; yield 74%. ^{1}H NMR (500 MHz, $CDCl_3$): δ =5.38 (m, 8H), 4.08 (t, J = 6.5 Hz, 4H), 2.32 (t, J = 7.4 Hz, 4H), 2.12–1.92 (m, 16H), 1.74–1.62 (m, 8H), 1.41–1.29 (m, 12H). ^{13}C NMR (126 MHz, $CDCl_3$): δ = 173.7, 130.2, 130.1, 129.3, 129.0, 64.4, 33.7, 29.5, 28.7, 28.6, 27.5, 27.3, 26.9, 26.5, 25.8, 24.9. ESI-MS: calcd. for $C_{32}H_{52}O_4$ + H^+ $[M + H]^+$ 501.3938; found 501.3953.

(7Z,11Z,25Z,29Z)-1,18-dioxacyclohexatriaconta-7,11,25,29-tetraene-2,17-dione (9c). Yellow oil; yield 80%. ^{1}H NMR (500 MHz, $CDCl_3$): δ = 5.43 – 5.35 (m, 8H), 4.08 (t, J = 6.4 Hz, 4H), 2.32 (t, J = 7.4 Hz, 4H), 2.09 – 1.94 (m, 16H), 1.72 – 1.56 (m, 8H), 1.43 – 1.36 (m, 16H). ^{13}C NMR (126 MHz, $CDCl_3$): δ = 173.8, 130.1, 129.7, 129.6, 129.3, 64.3, 34.3, 29.5, 29.1, 28.8, 28.6, 27.5, 27.4, 27.0, 26.8, 25.8, 24.7. ESI-MS: calcd. for $C_{34}H_{56}O_4$ + Na^+ $[M + Na]^+$ 551.4071; found 551.4107.

(8Z,12Z,27Z,31Z)-1,20-dioxacyclooctatriaconta-8,12,27,31-tetraene-2,19-dione (9d). Yellow oil; yield 79%. ^{1}H NMR (500 MHz, $CDCl_3$): δ = 5.46 – 5.33 (m, 8H), 4.08 (t, J = 6.5 Hz, 4H), 2.31 (t, J = 7.4 Hz, 4H), 2.09 – 1.92 (m, 16H), 1.68 – 1.60 (m, 8H), 1.44 – 1.28 (m, 20H). ^{13}C NMR (126 MHz, $CDCl_3$): δ = 173.9, 130.1, 129.9, 129.4, 129.3, 64.3, 34.4, 29.5, 29.3, 28.8, 28.7, 28.6, 27.5, 27.4, 27.0, 26.9, 25.8, 24.9. ESI-MS: calcd. for $C_{36}H_{60}O_4$ + Na^+ $[M + Na]^+$ 579,4384; found 579,4298.

^{1}H NMR and ^{13}C NMR spectra of compounds 6a-9d.

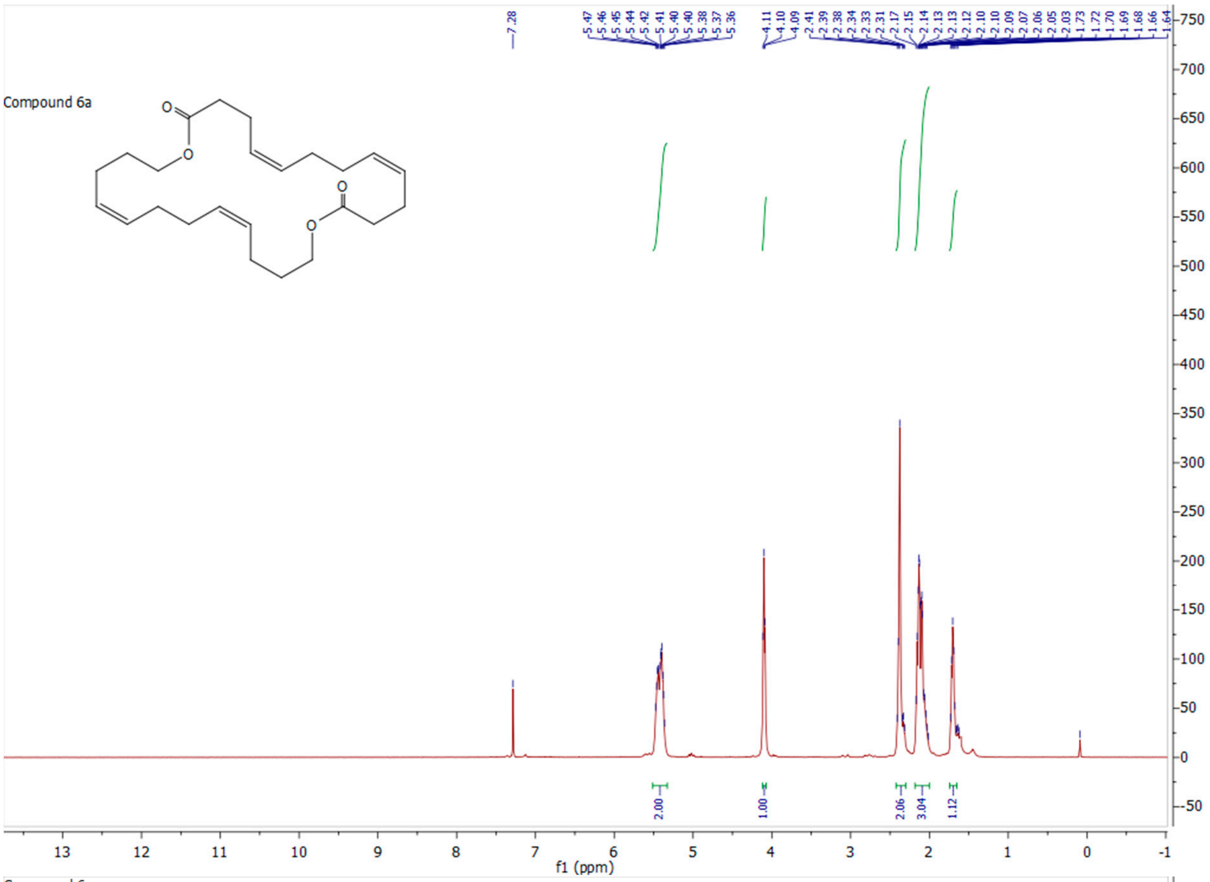

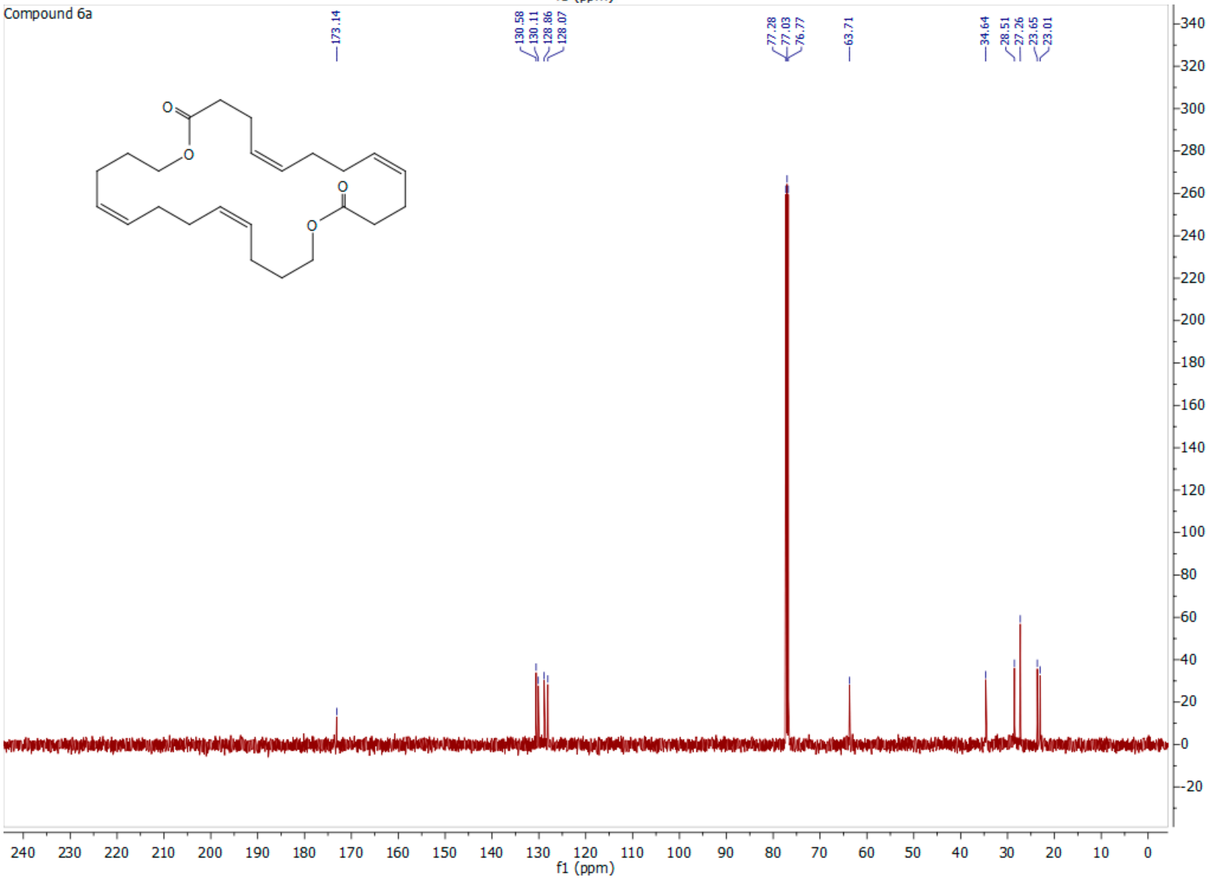

Figure S1. 1H NMR and 13C NMR spectra of compound 6a.

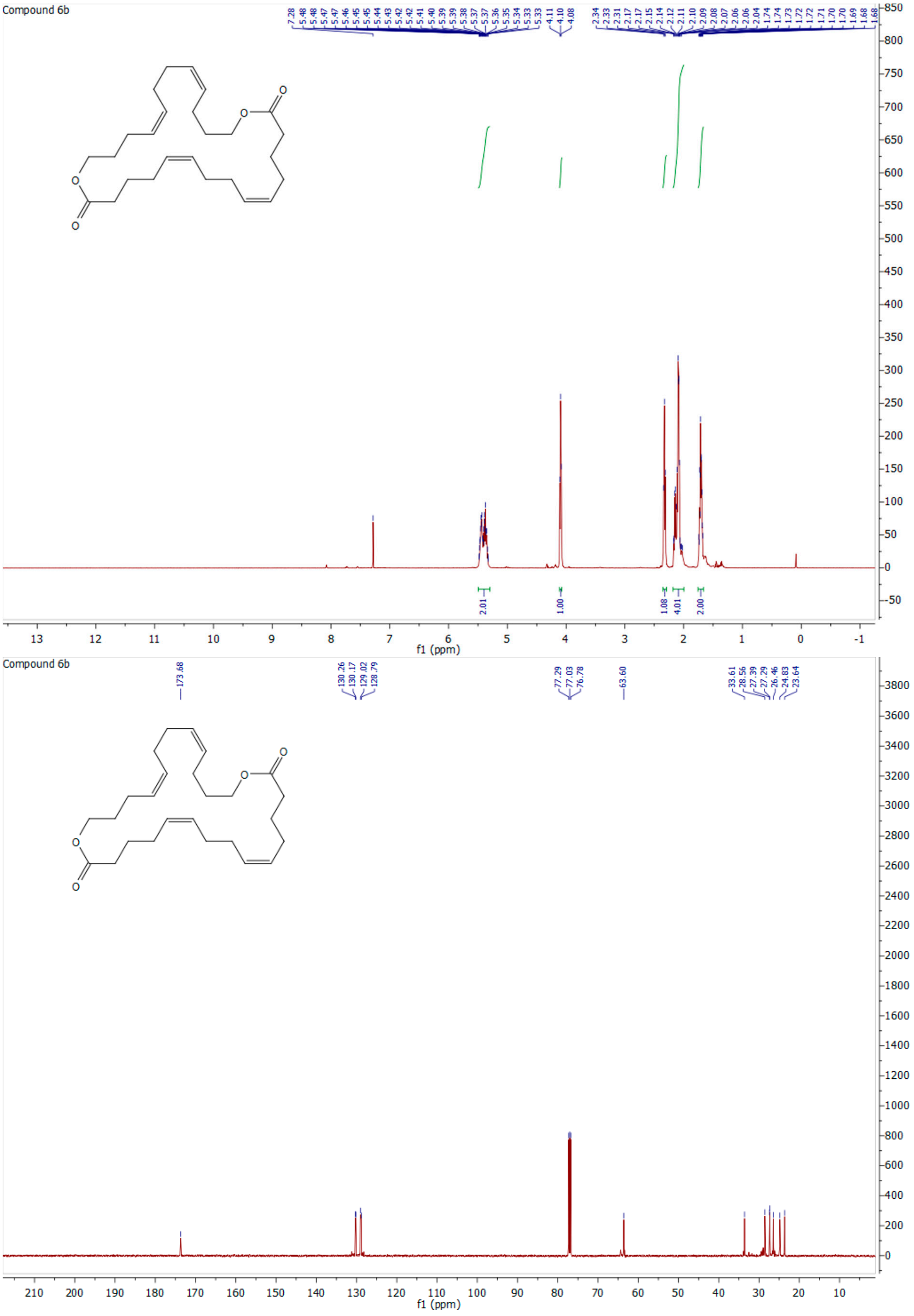

Figure S2. 1H NMR and 13C NMR spectra of compound 6b.

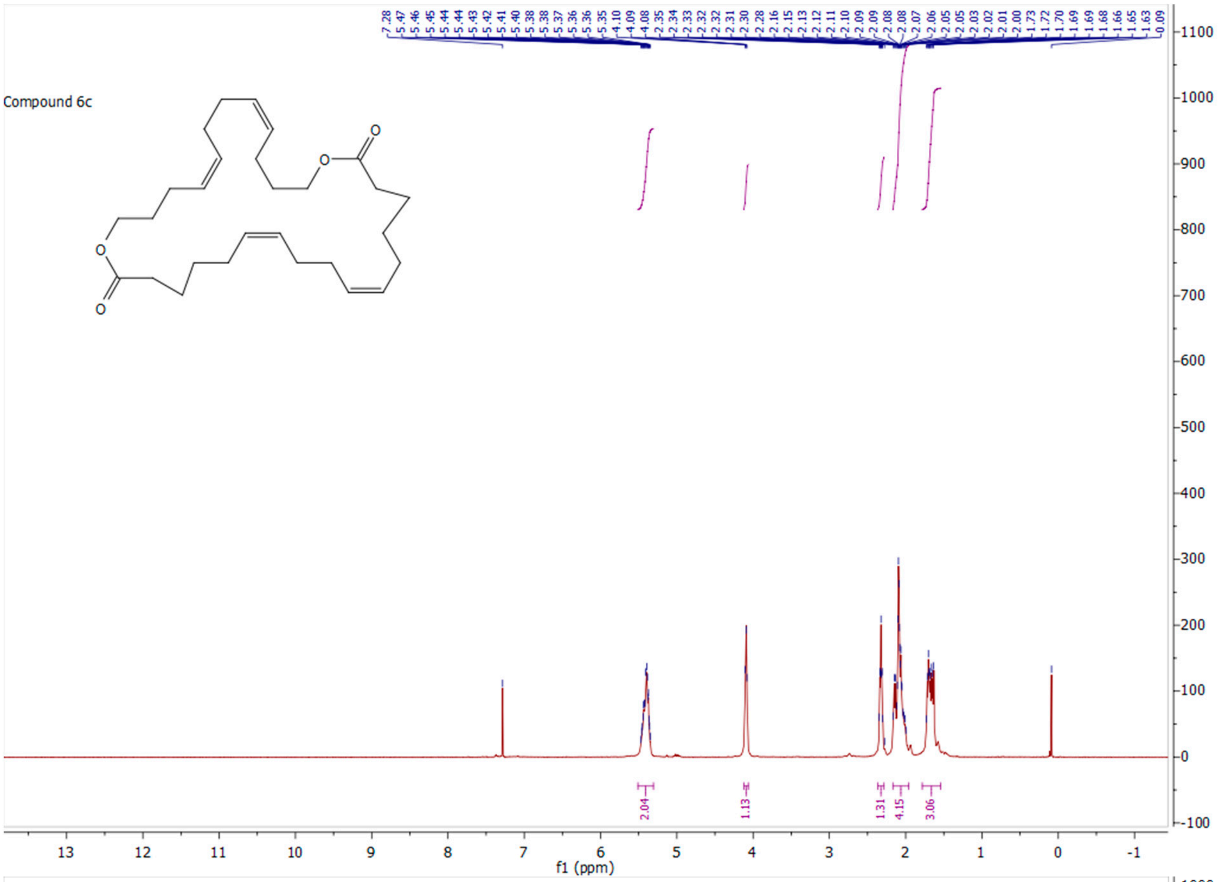

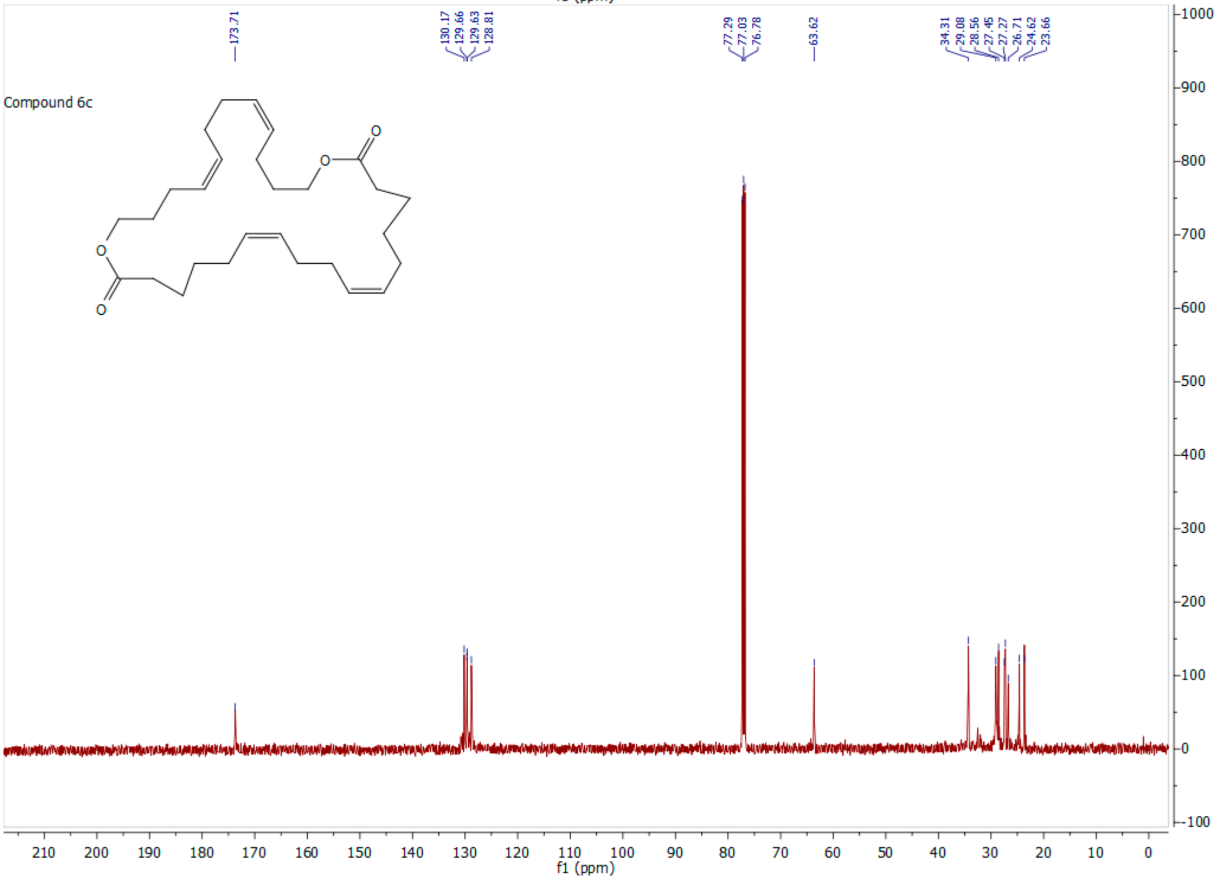

Figure S3. 1H NMR and 13C NMR spectra of compound 6c.

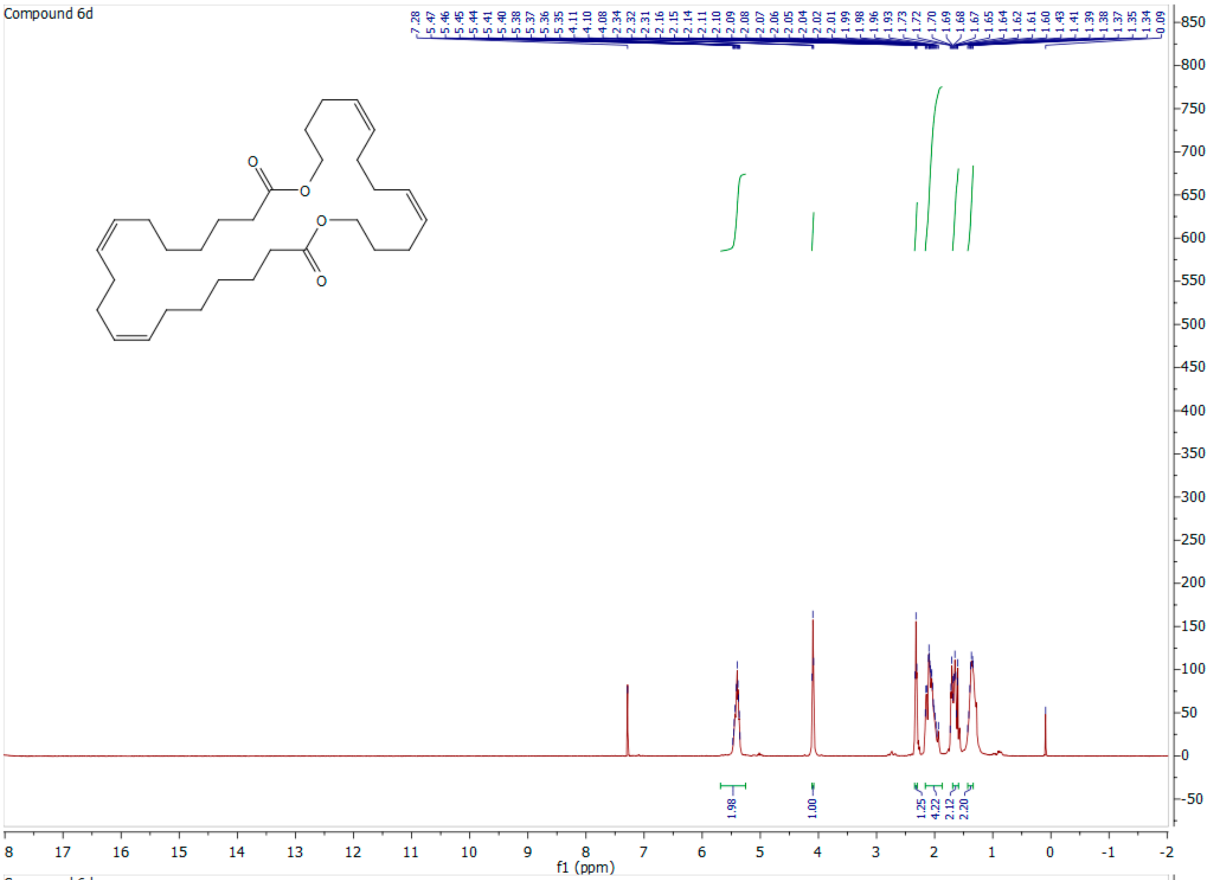

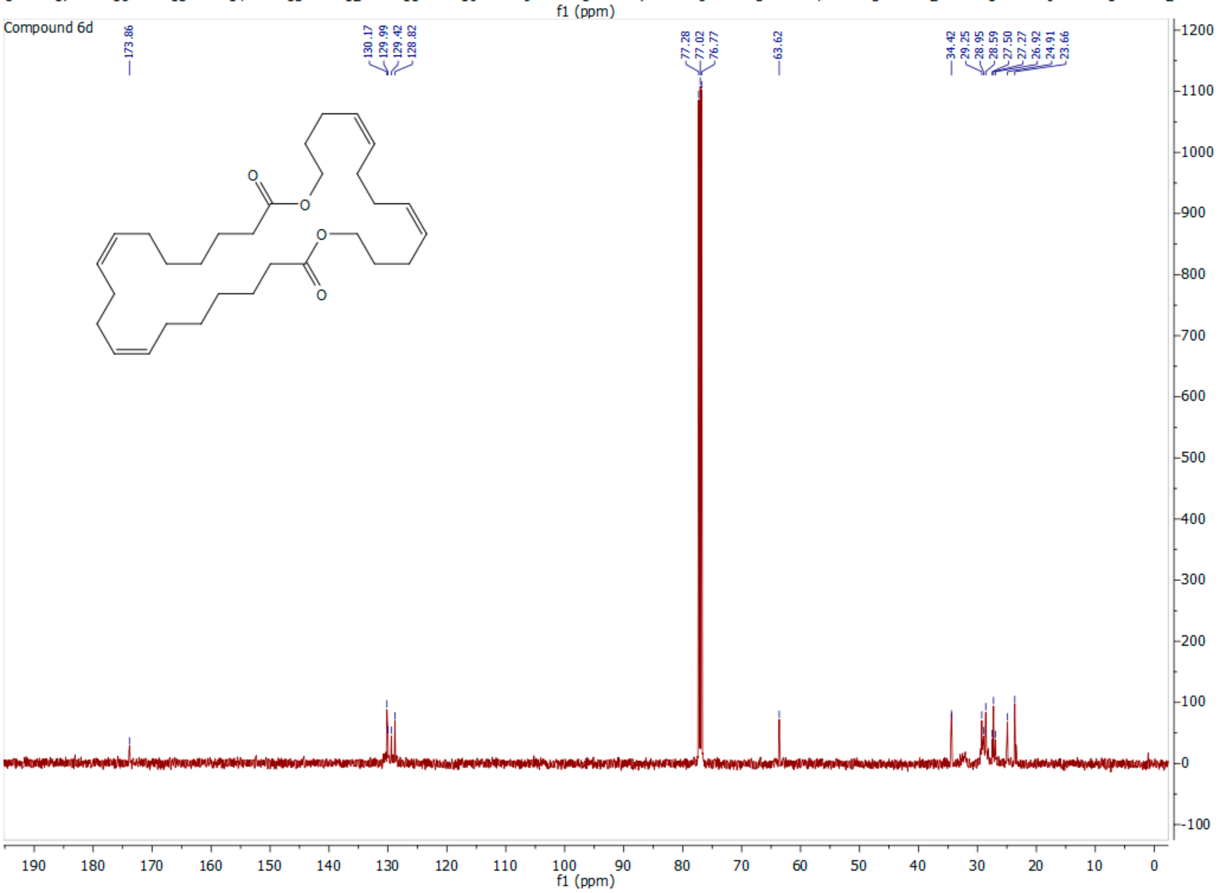

Figure S4. 1H NMR and 13C NMR spectra of compound 6d.

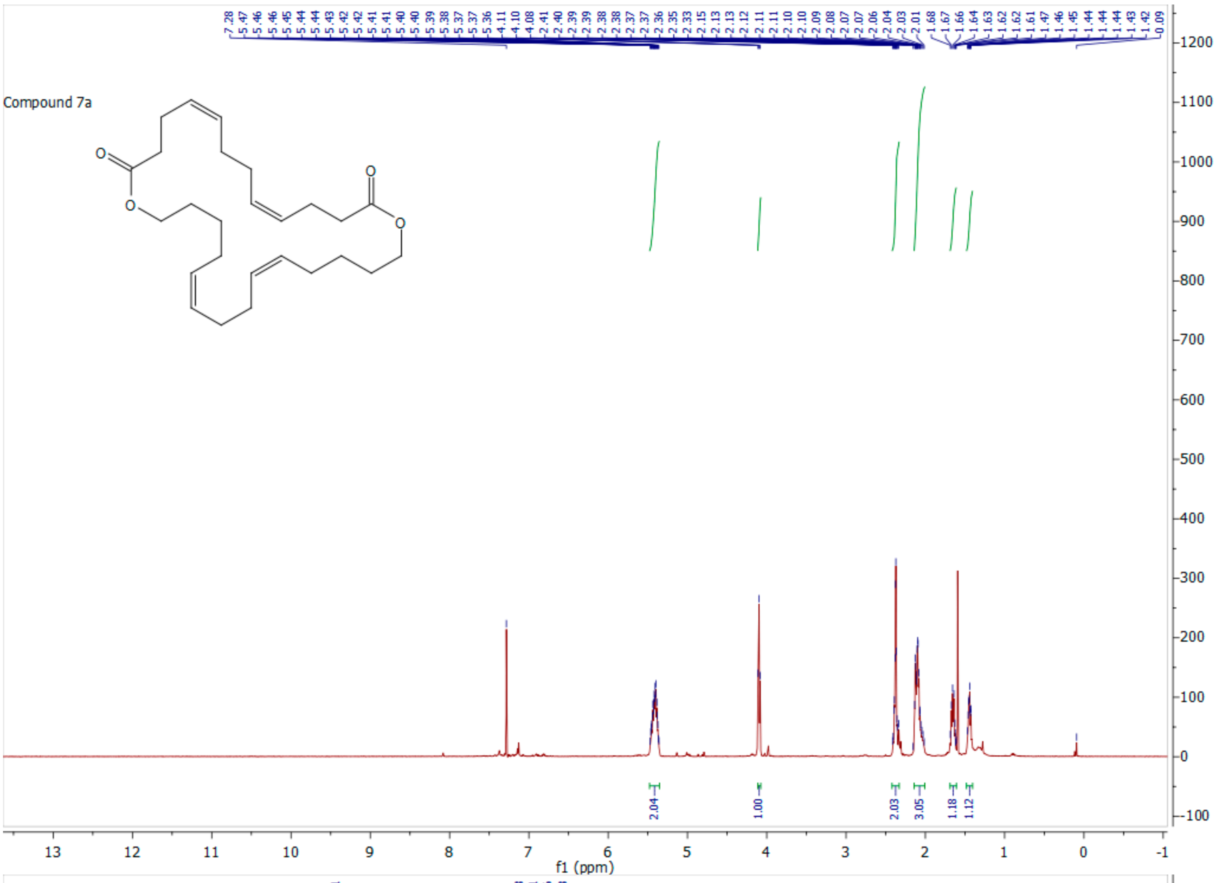

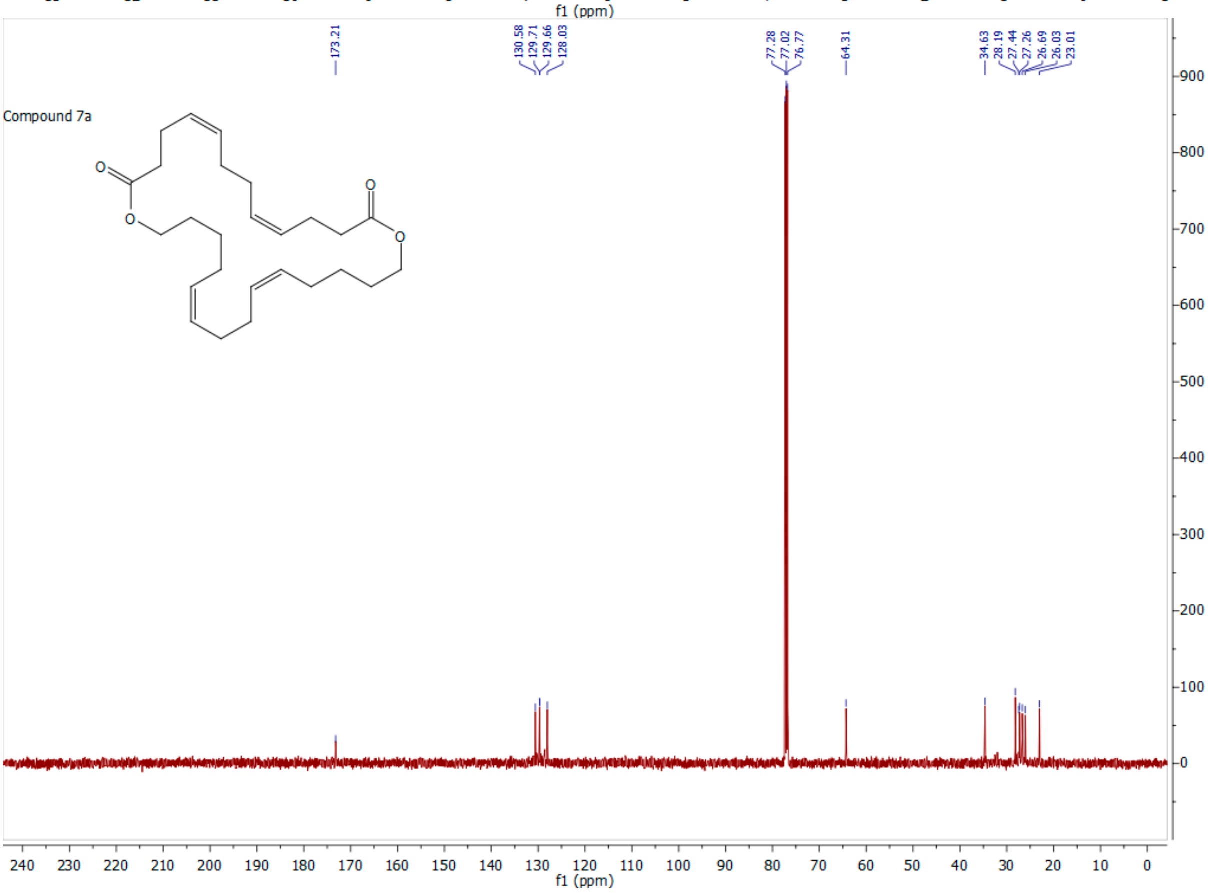

Figure S5. 1H NMR and 13C NMR spectra of compound 7a.

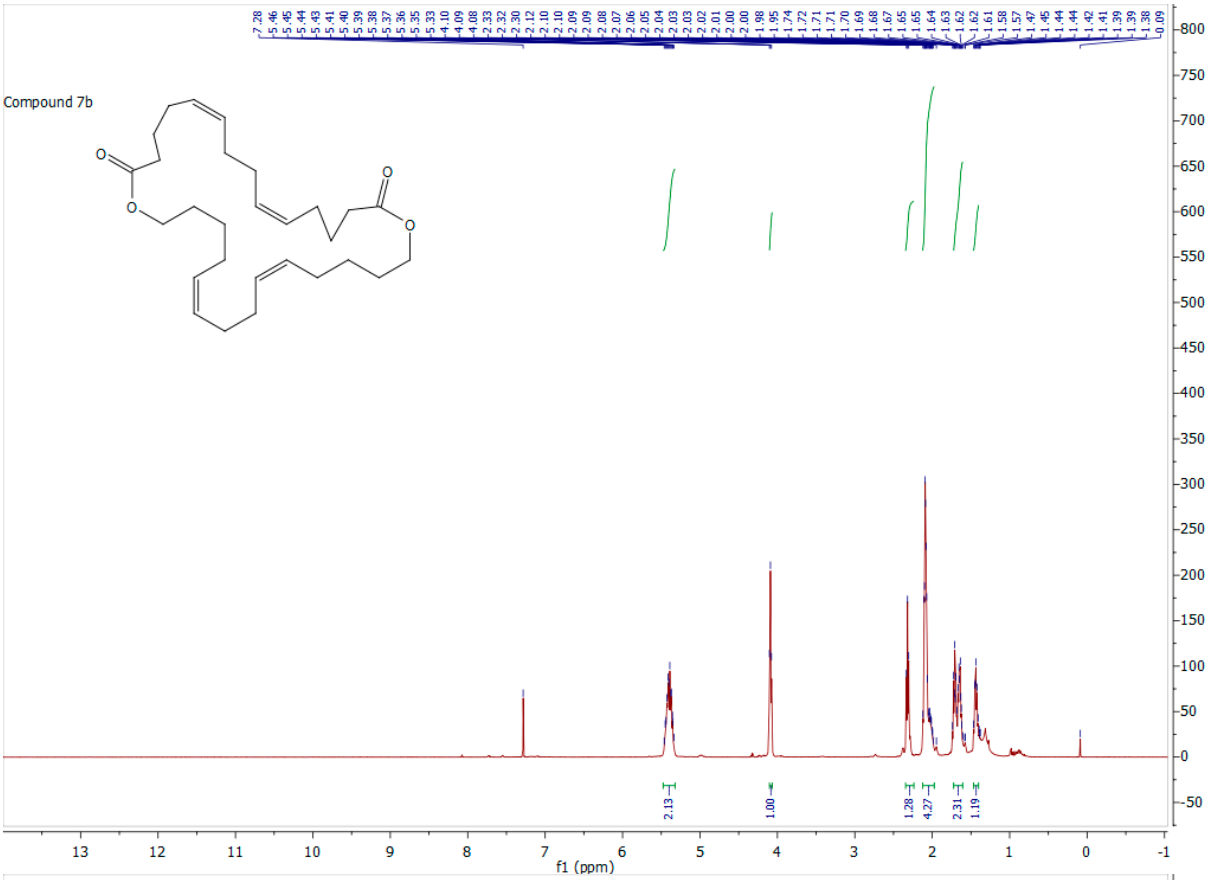

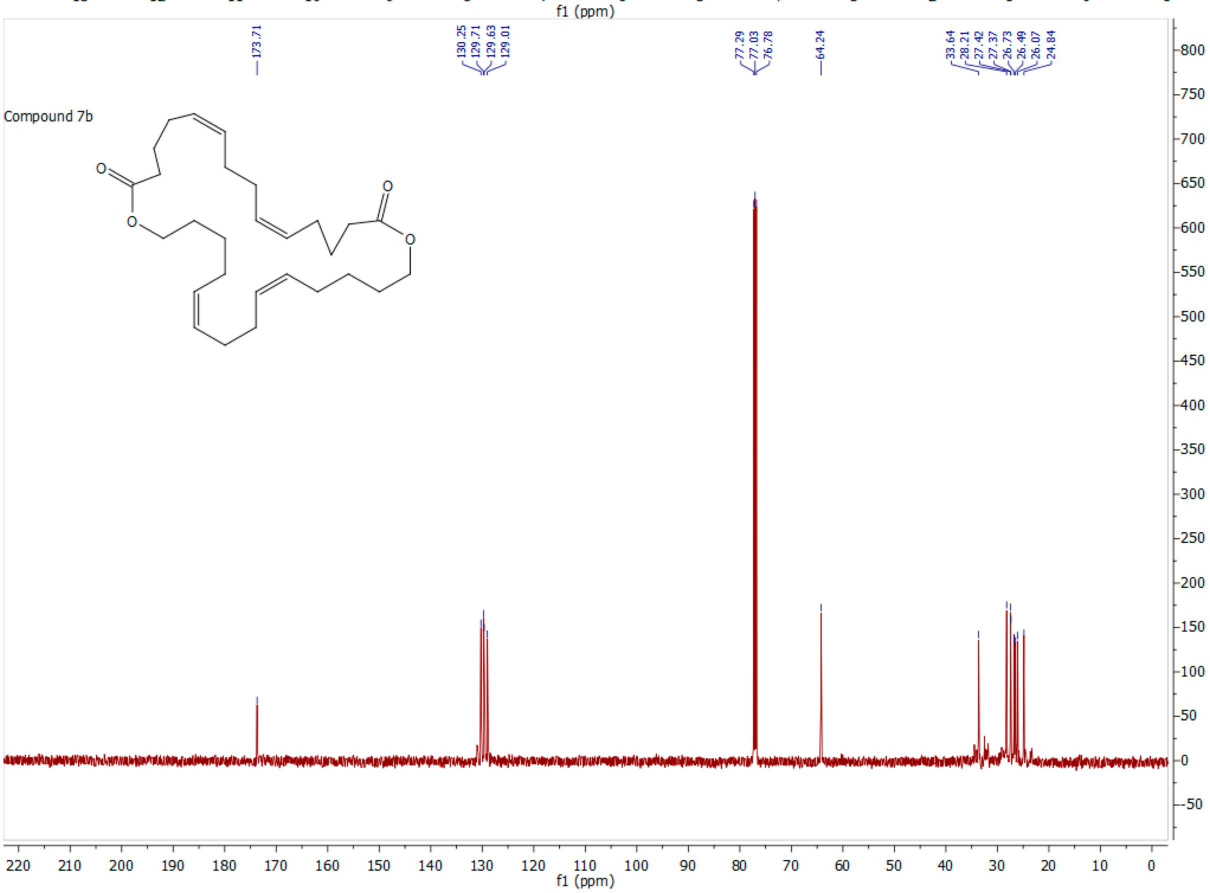

Figure S6. 1H NMR and 13C NMR spectra of compound 7b.

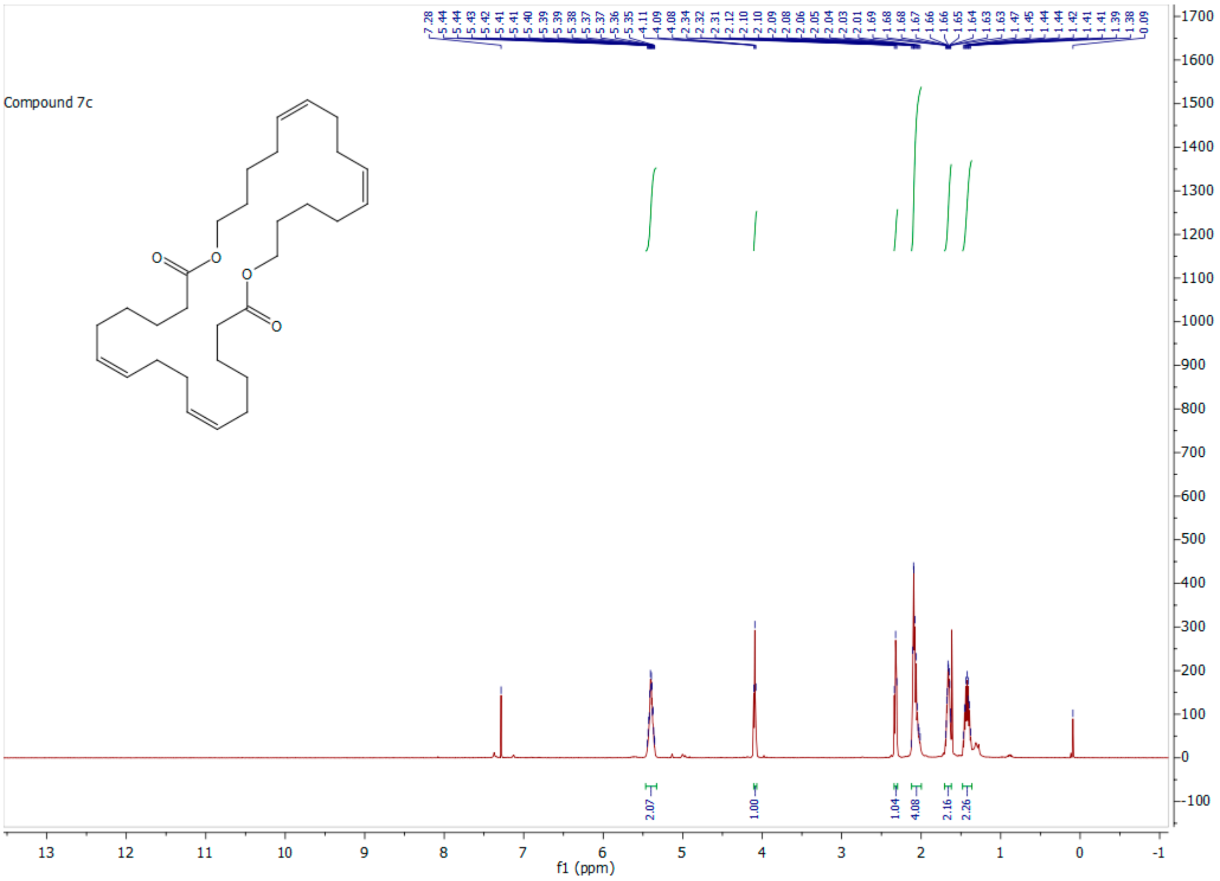

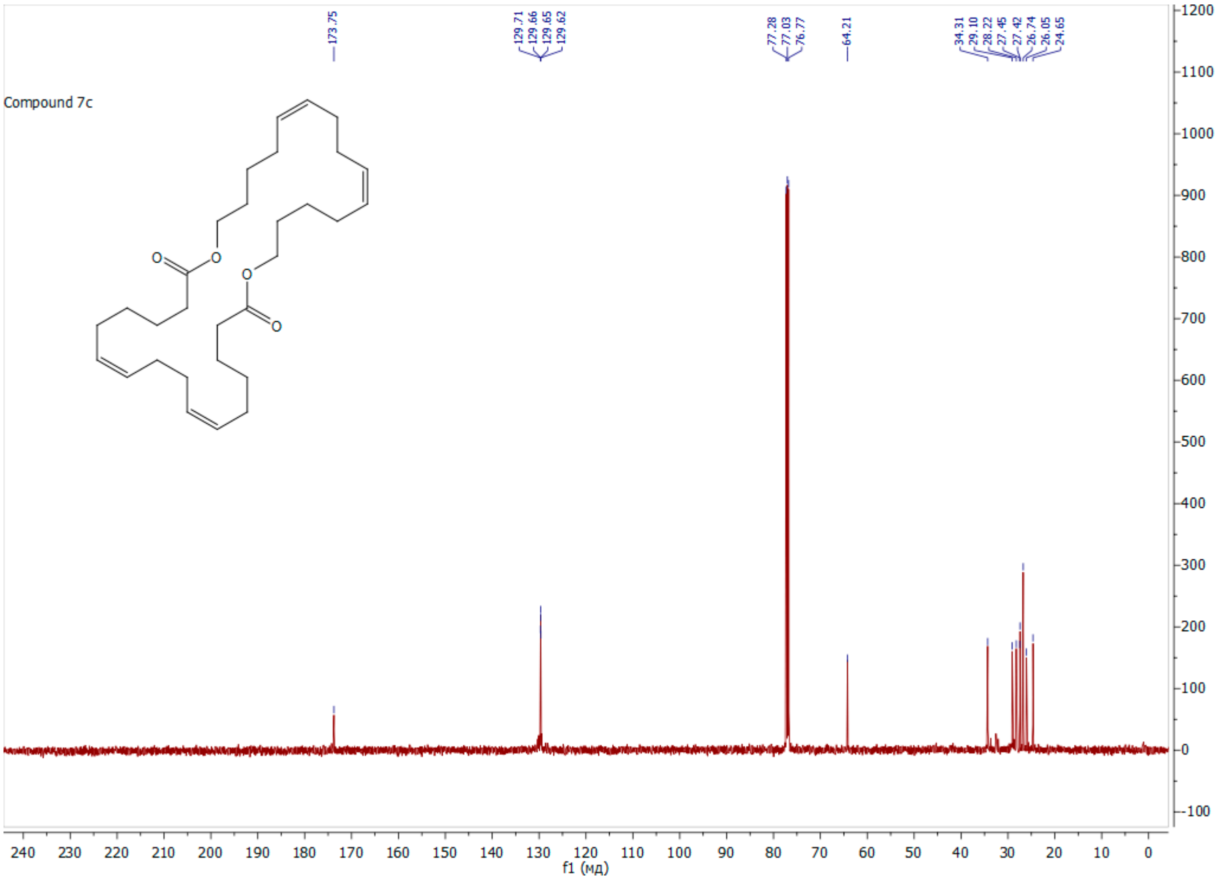

Figure S7. 1H NMR and 13C NMR spectra of compound 7c.

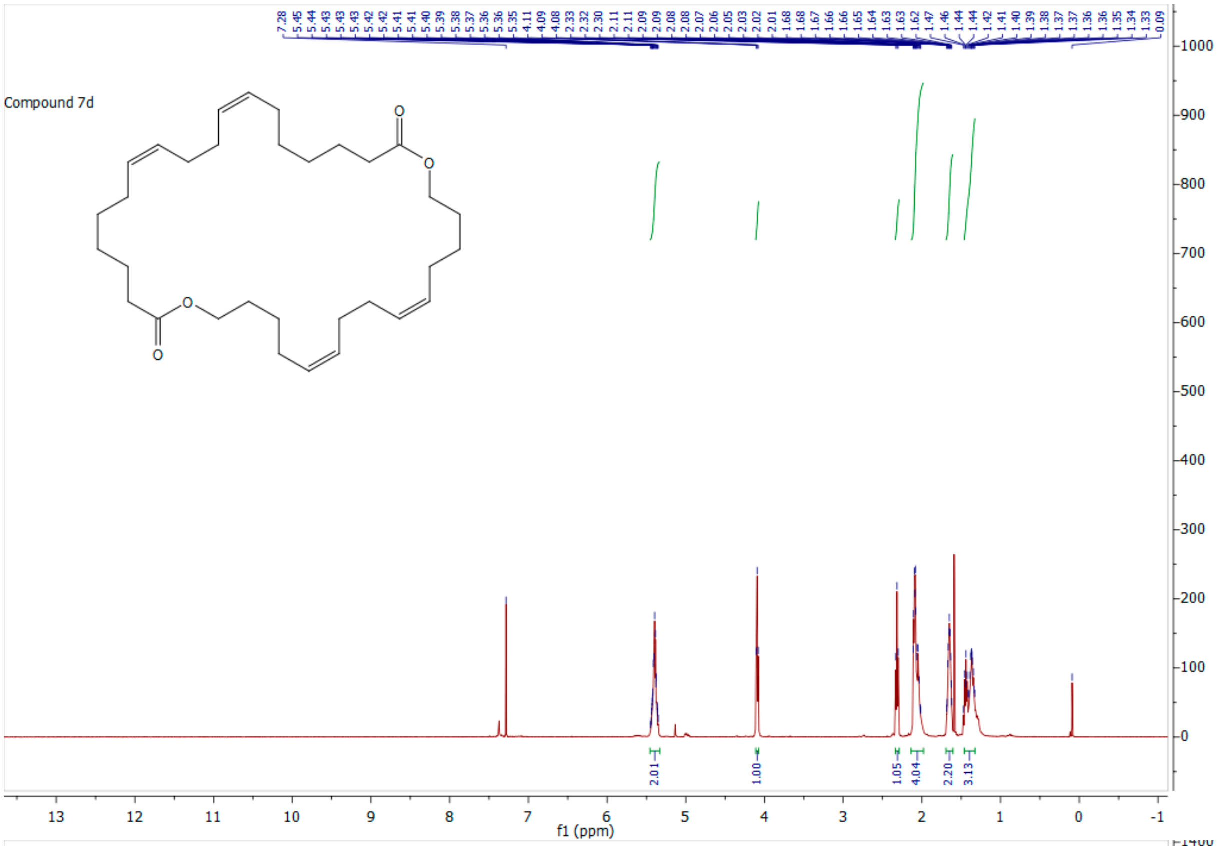

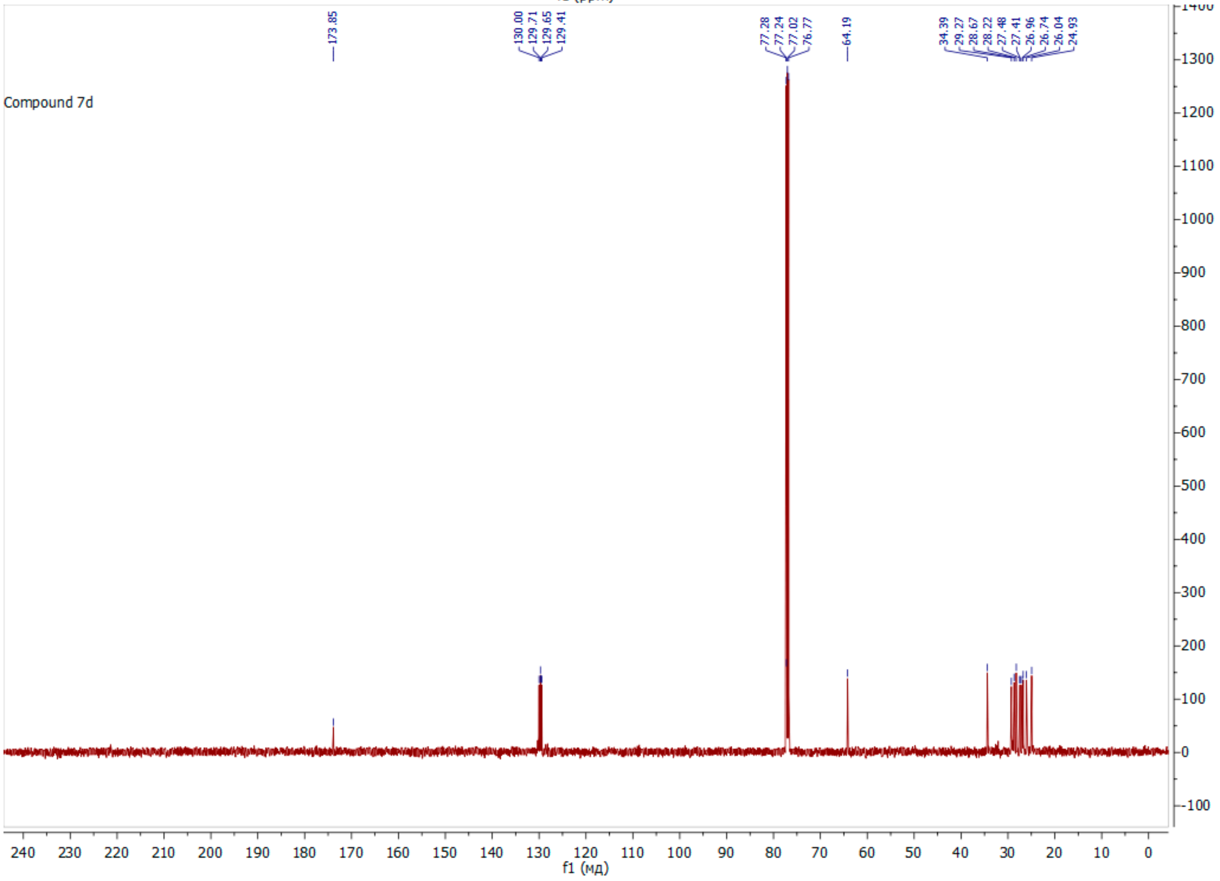

Figure S8. 1H NMR and 13C NMR spectra of compound 7d.

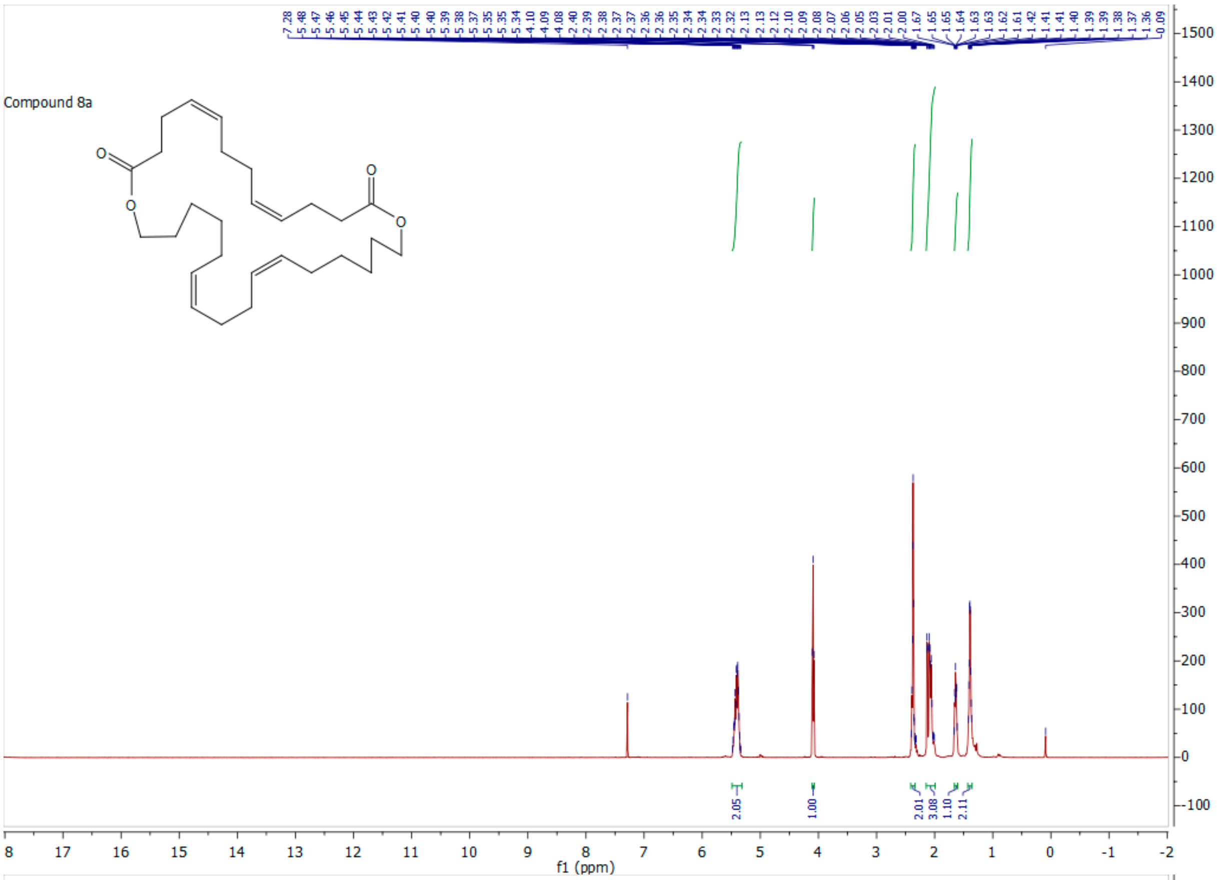

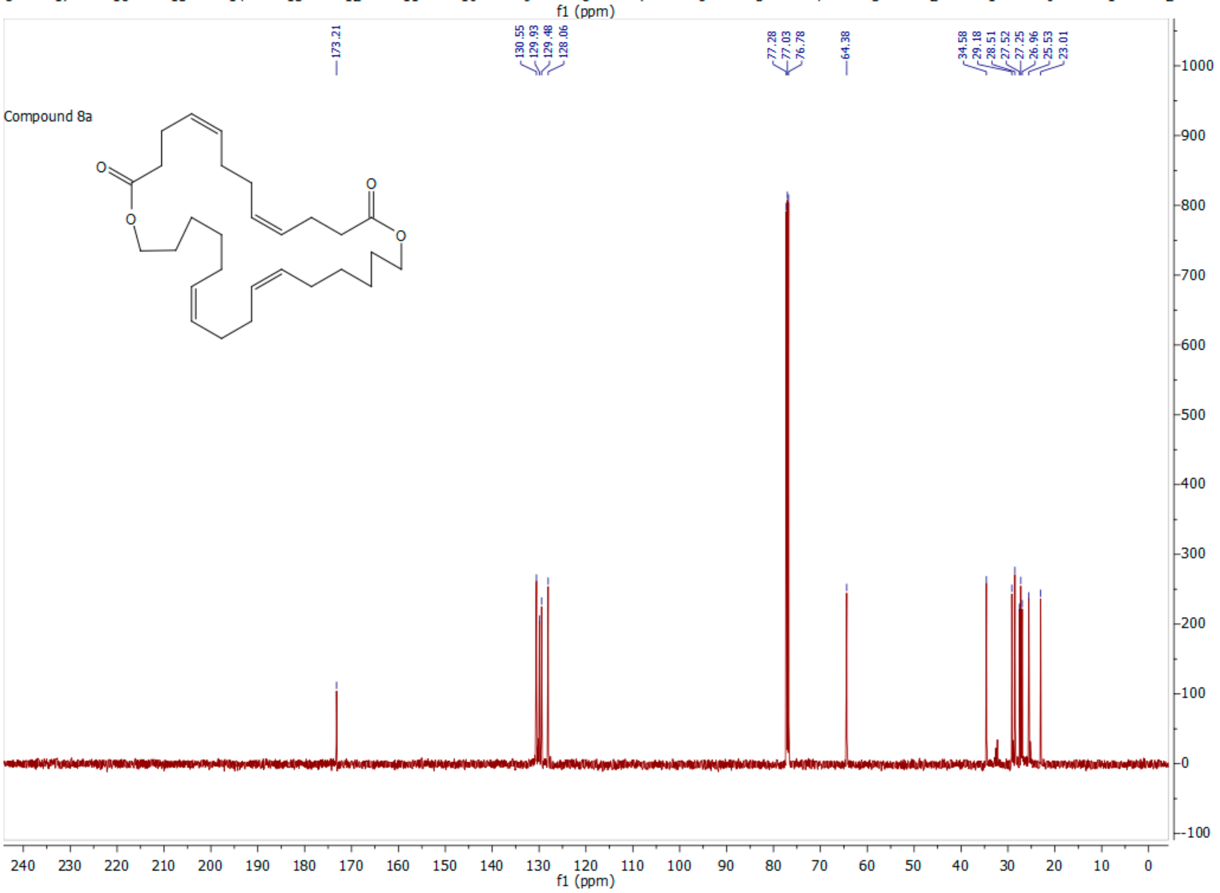

Figure S9. 1H NMR and 13C NMR spectra of compound 8a.

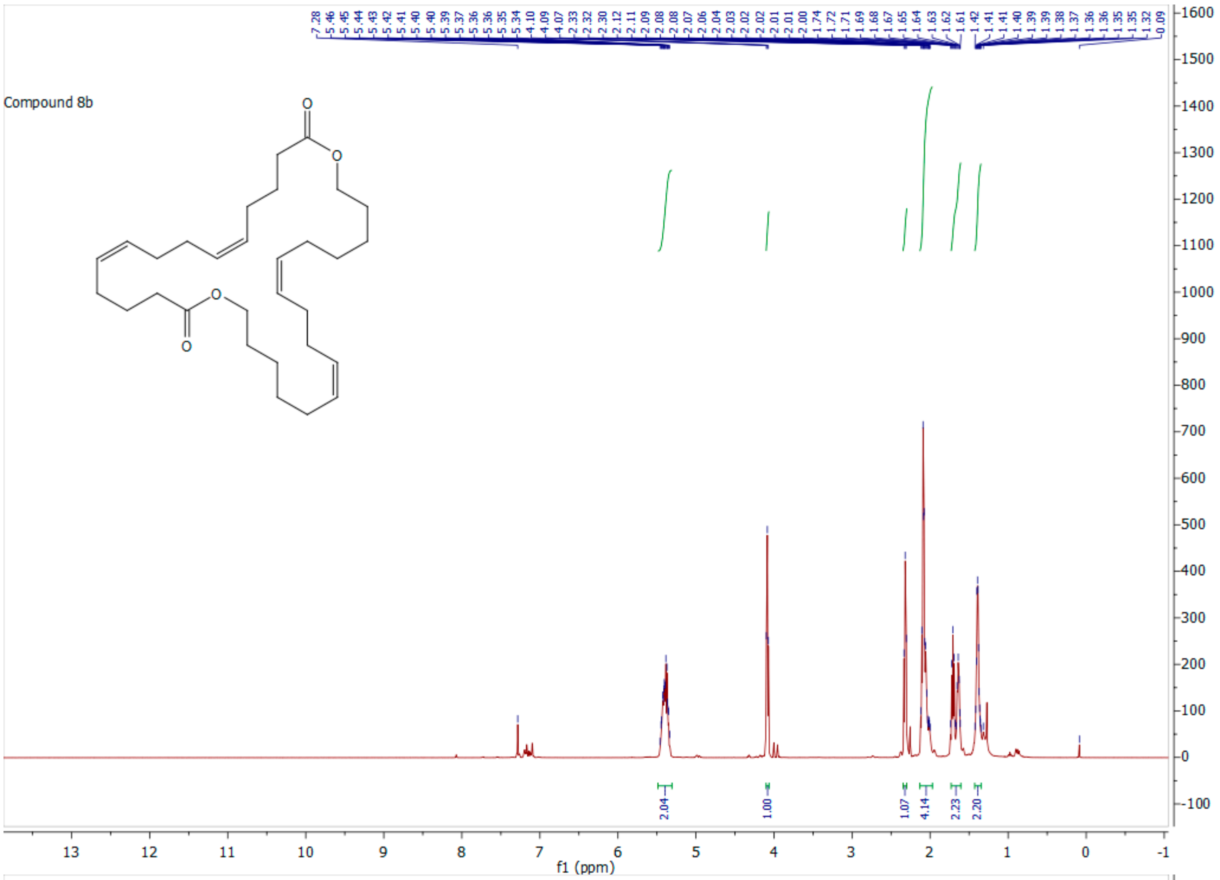

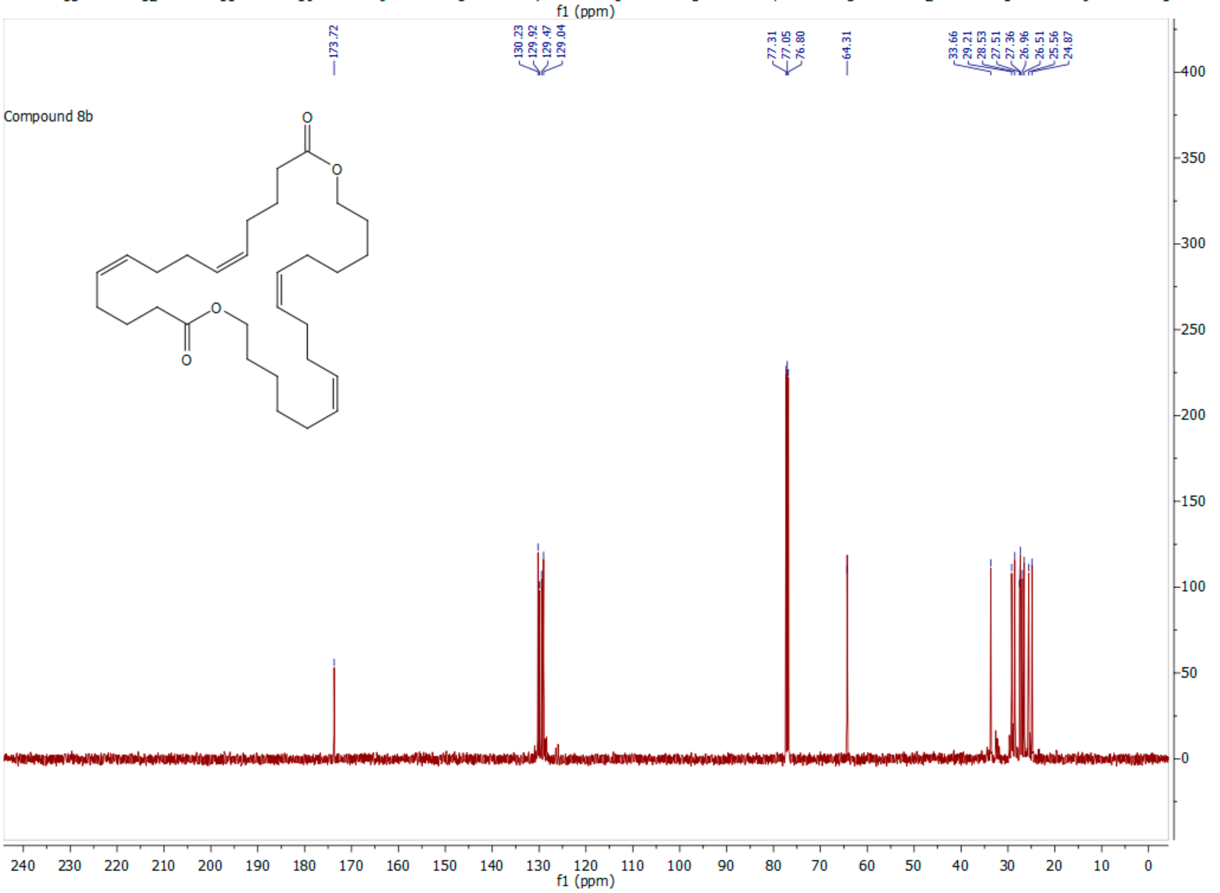

Figure S10. 1H NMR and 13C NMR spectra of compound 8b.

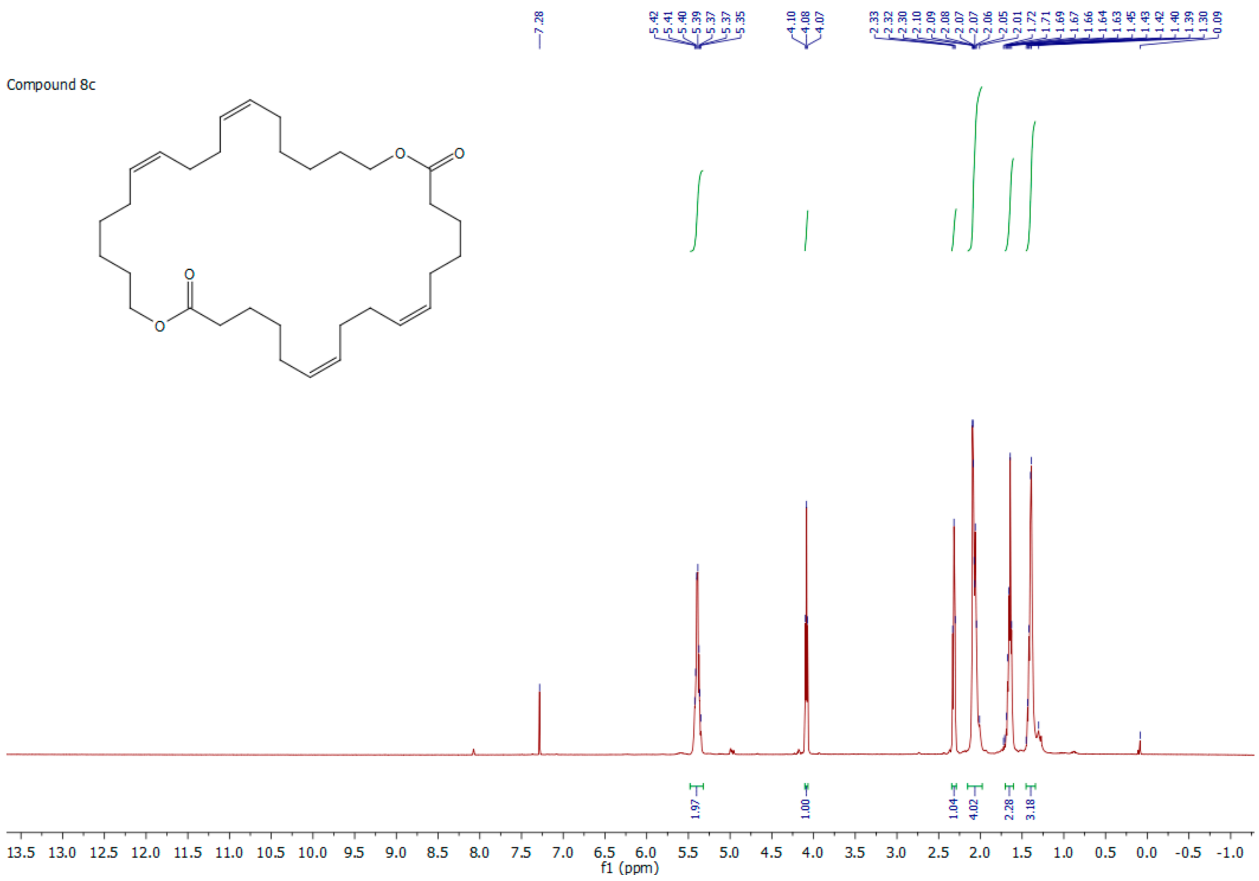

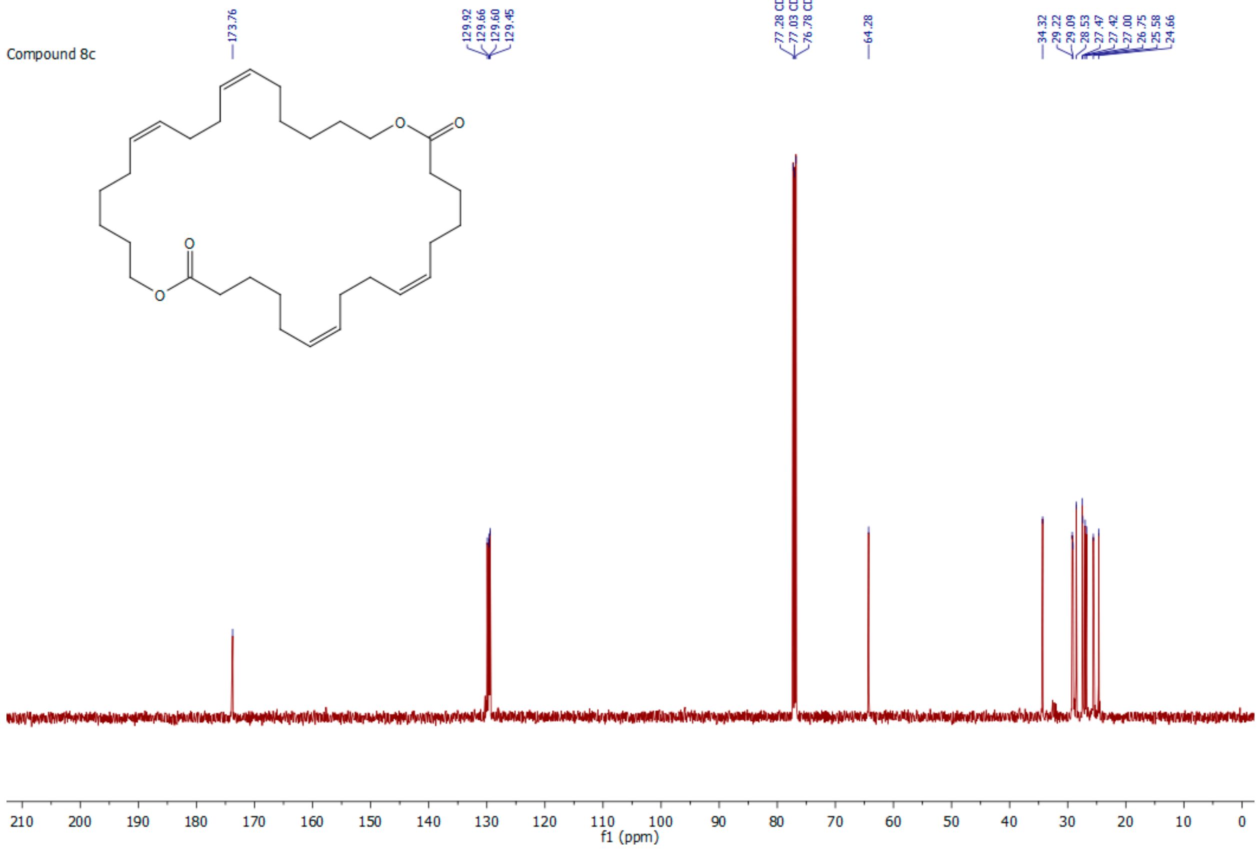

Figure S11. 1H NMR and 13C NMR spectra of compound 8c.

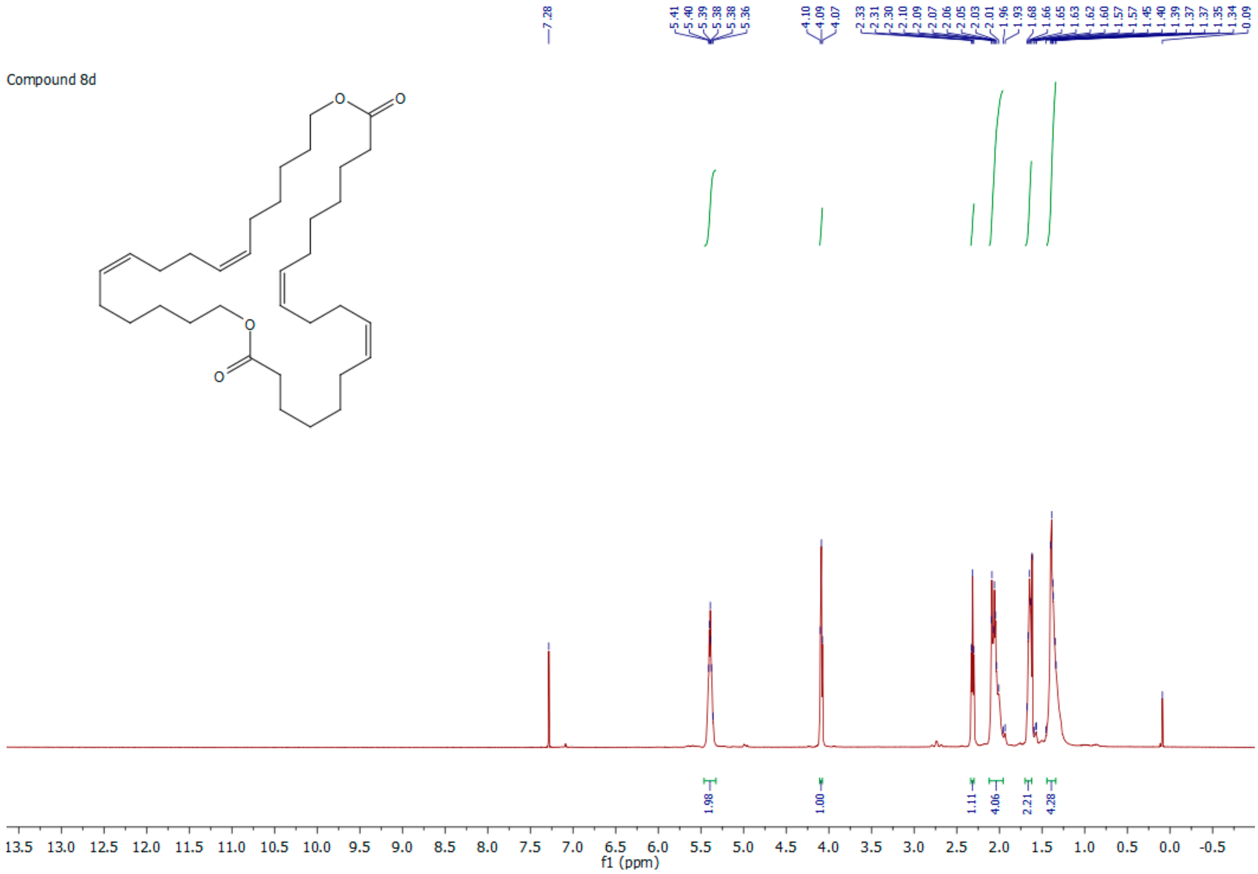

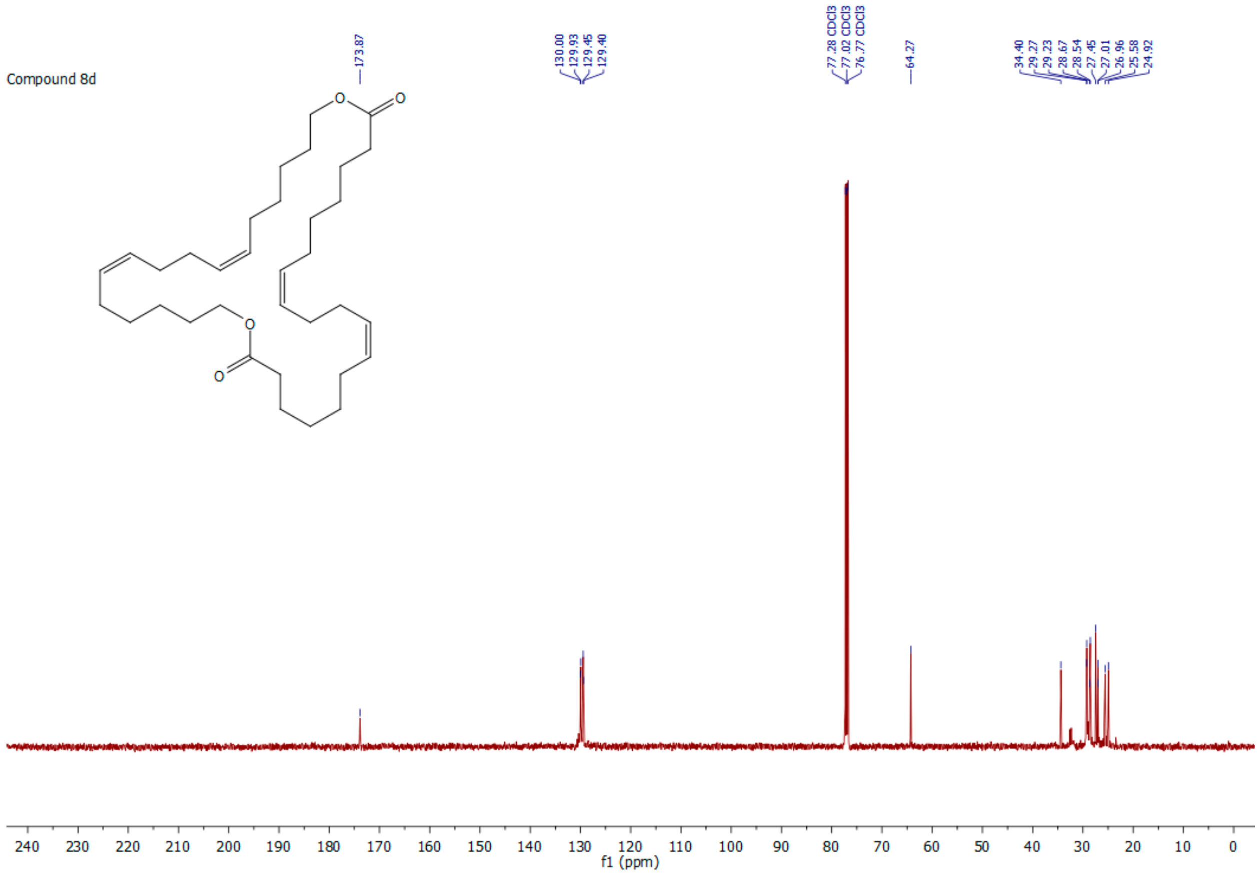

Figure S12. 1H NMR and 13C NMR spectra of compound 8d.

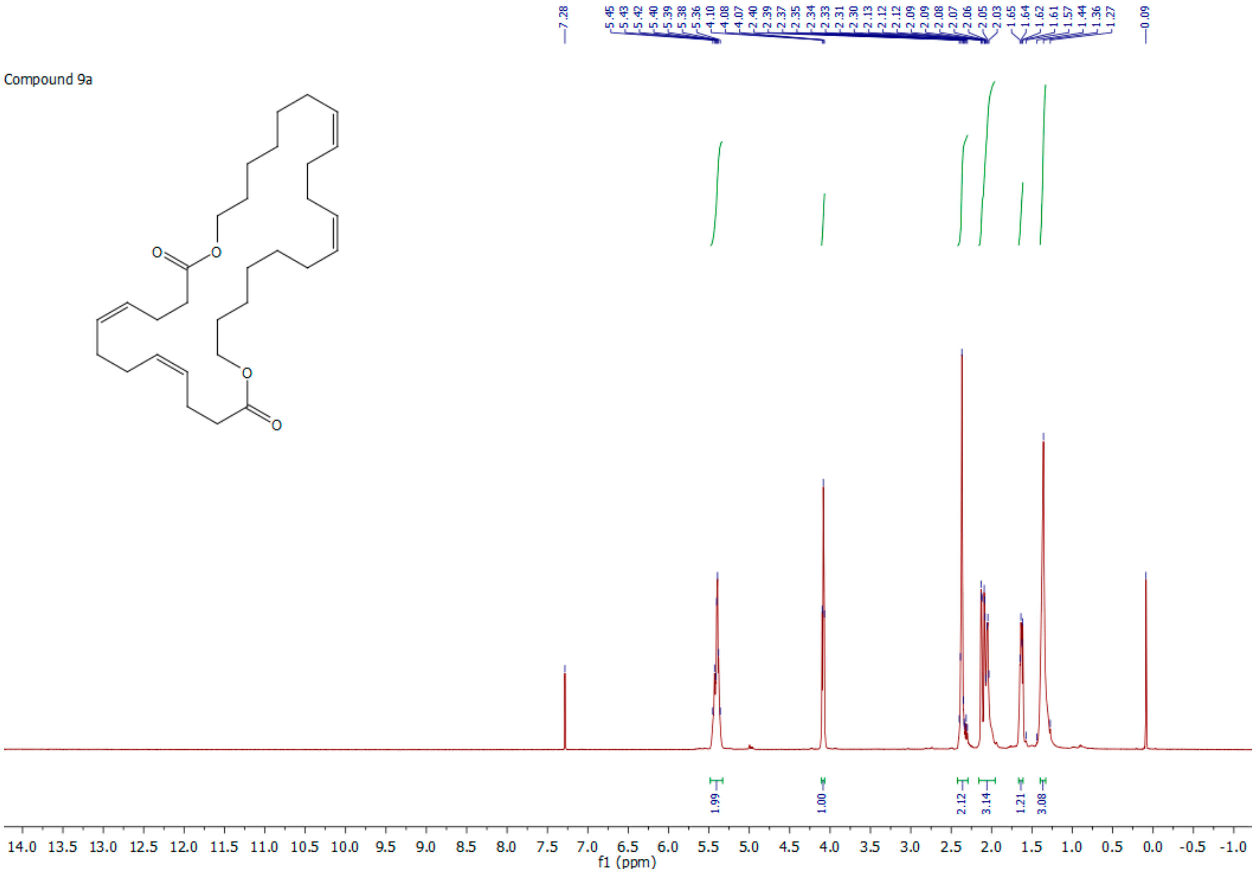

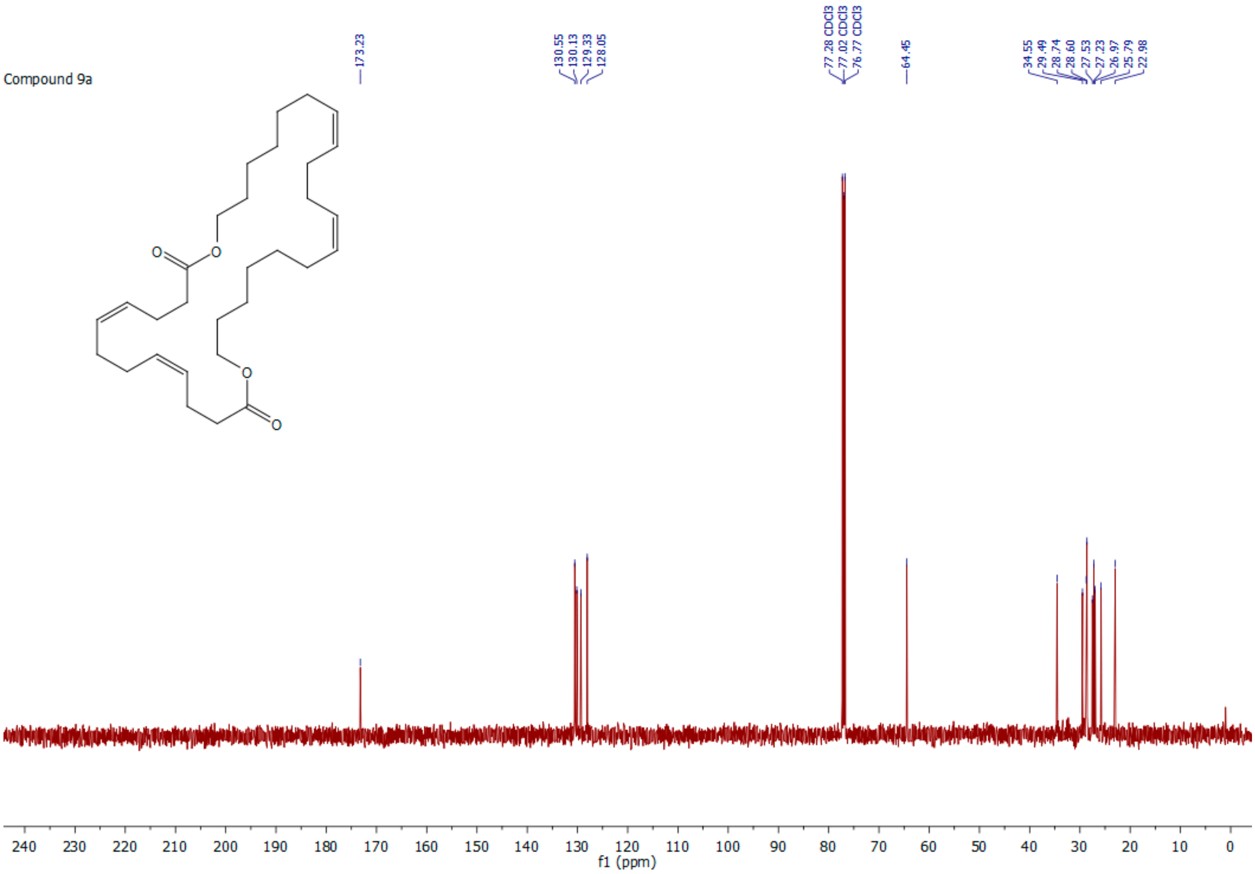

Figure S13. 1H NMR and 13C NMR spectra of compound 9a.

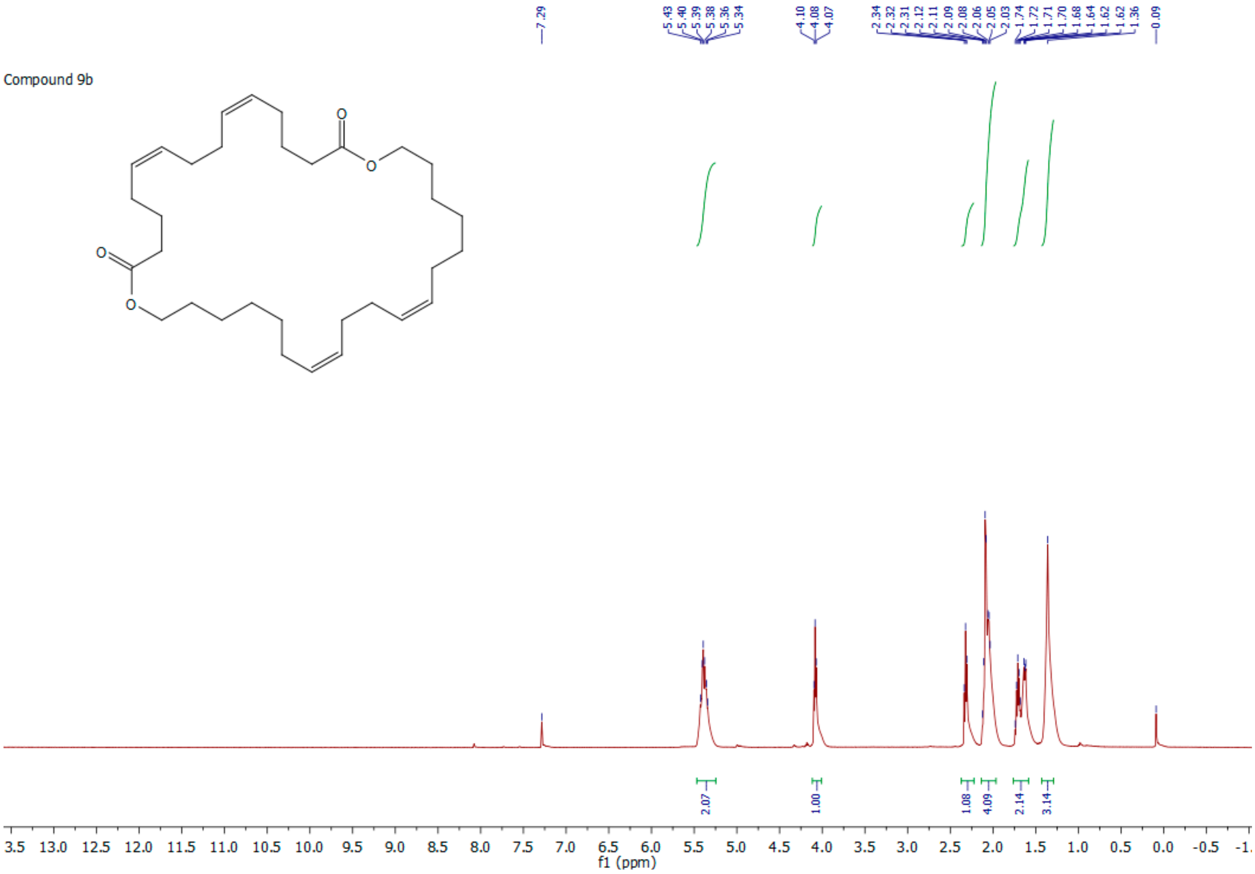

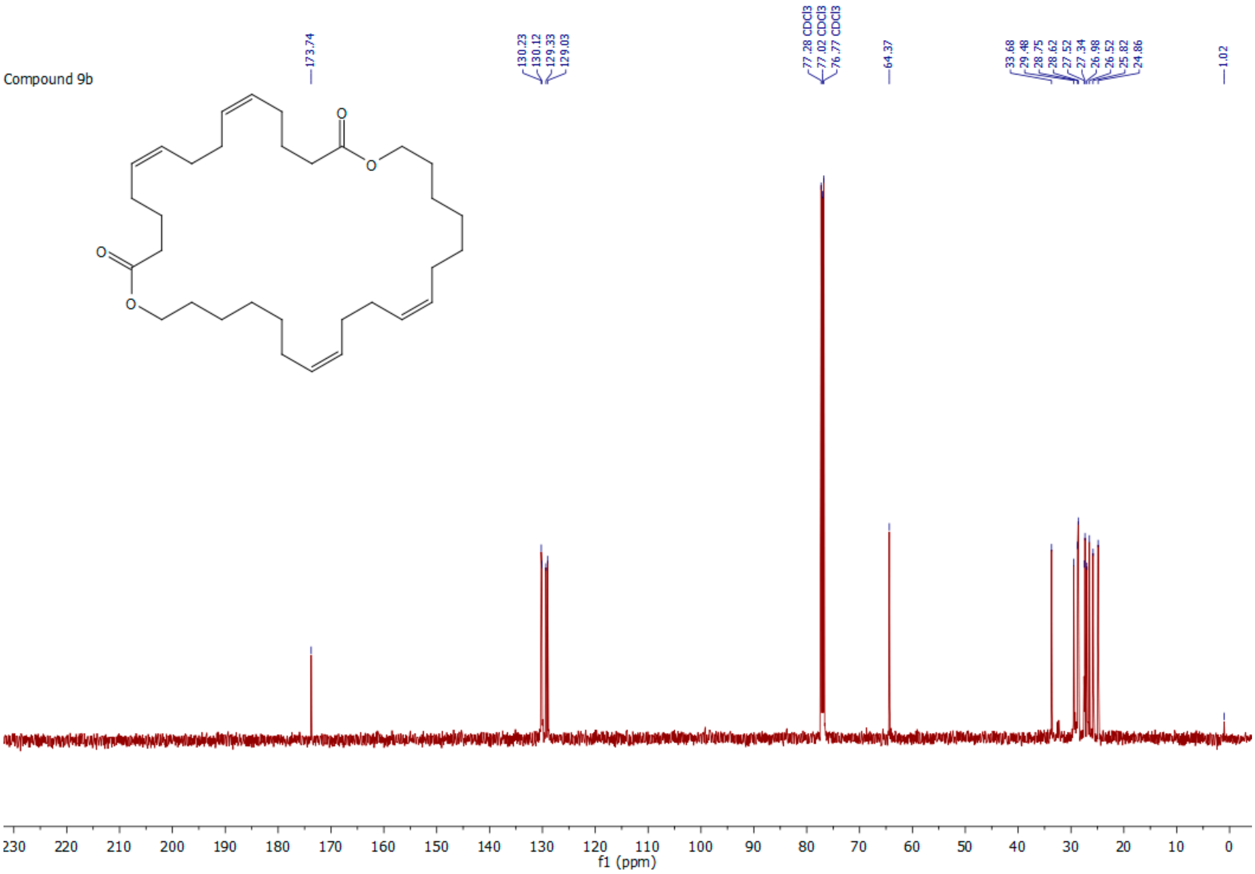

Figure S14. 1H NMR and 13C NMR spectra of compound 9b.

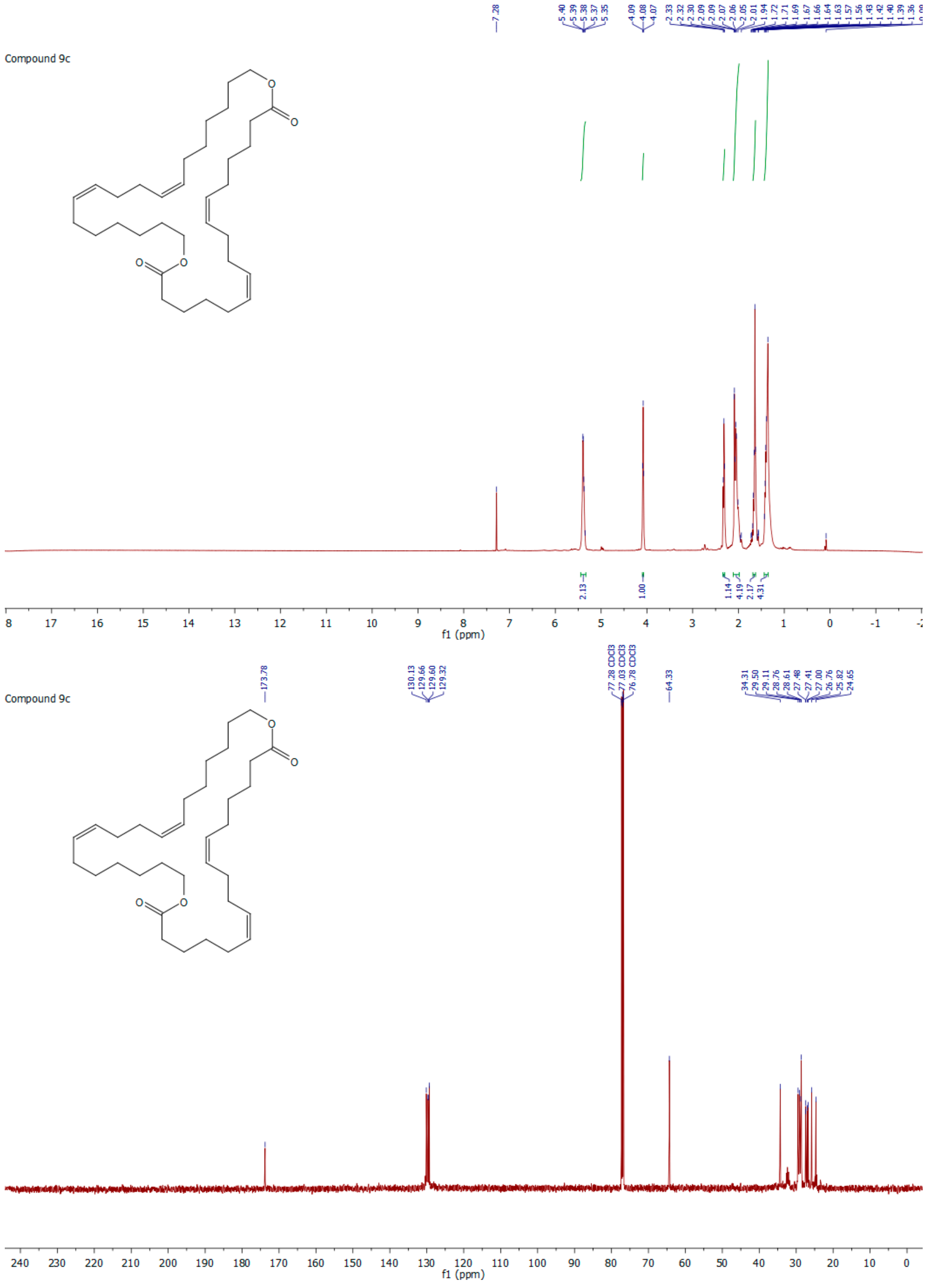

Figure S15. 1H NMR and 13C NMR spectra of compound 9c.

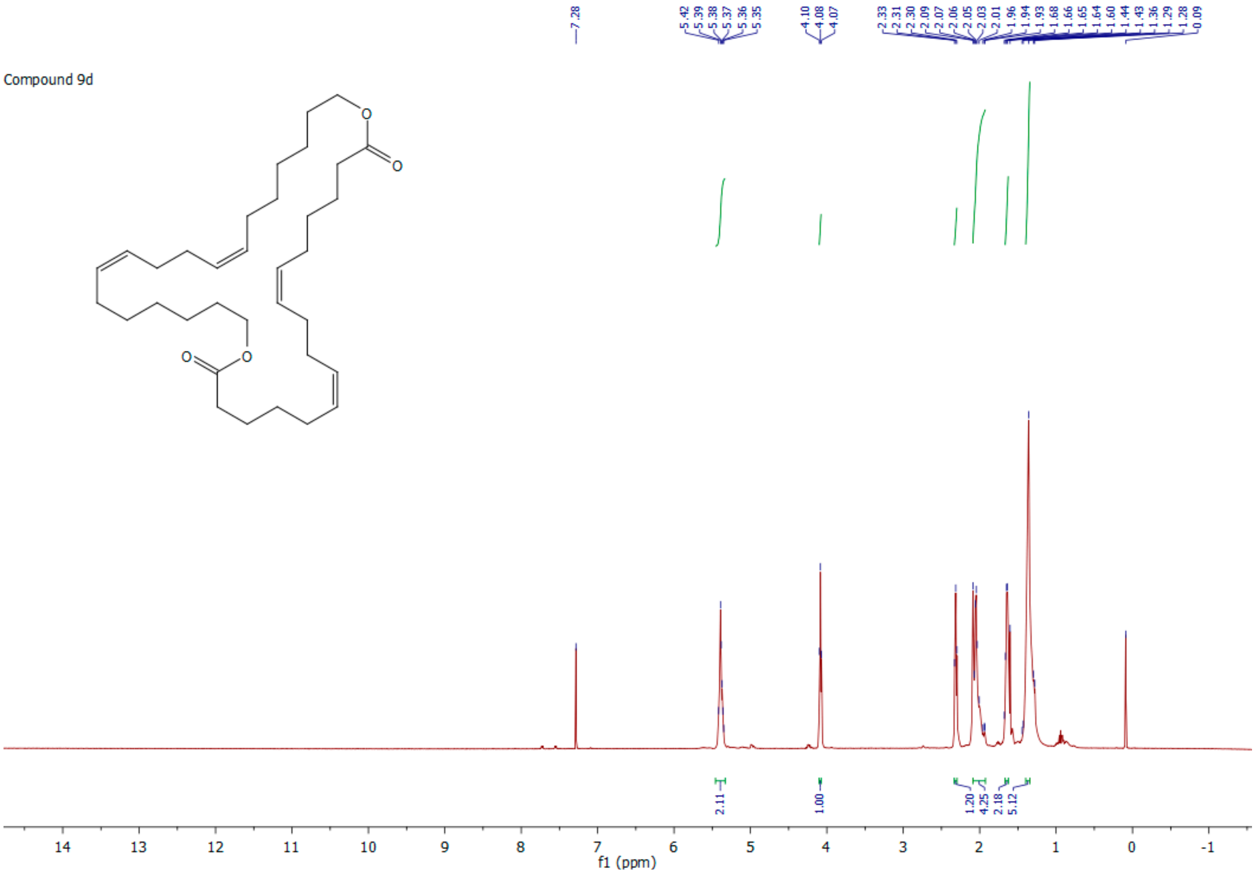

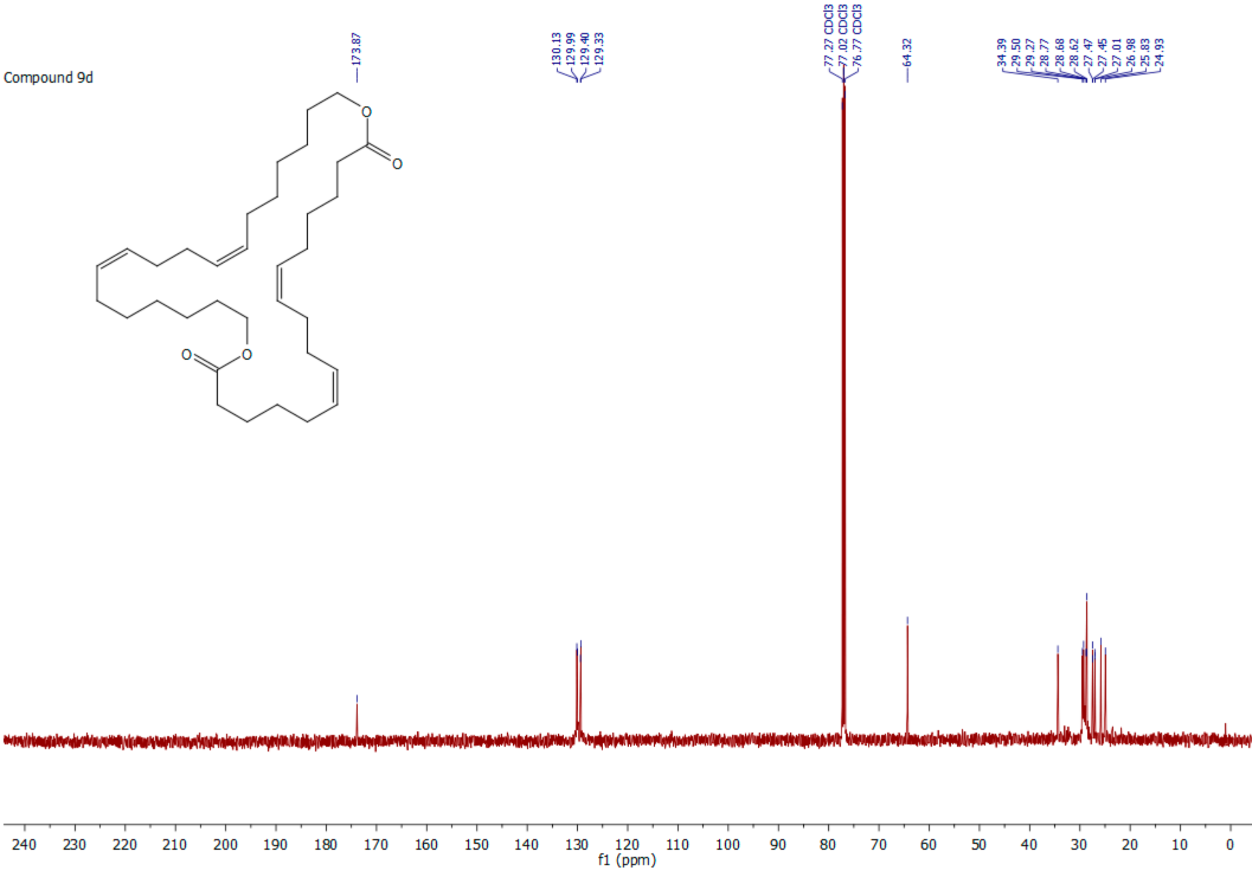

Figure S16. 1H NMR and 13C NMR spectra of compound 9d.

Table S1. Detecrion of changes in mitochondrial membrane potential (ΔΨ) and the associated early and late stages

contr
ol

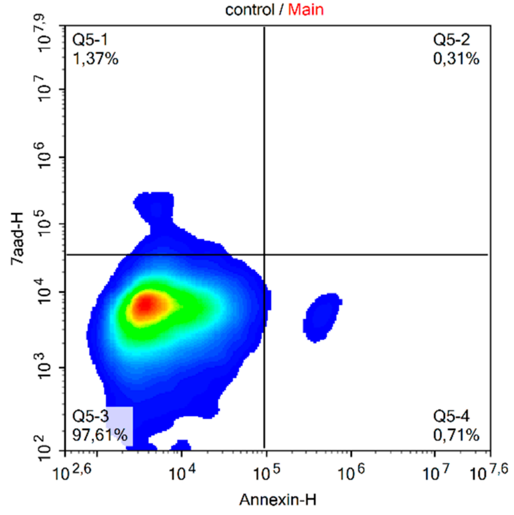

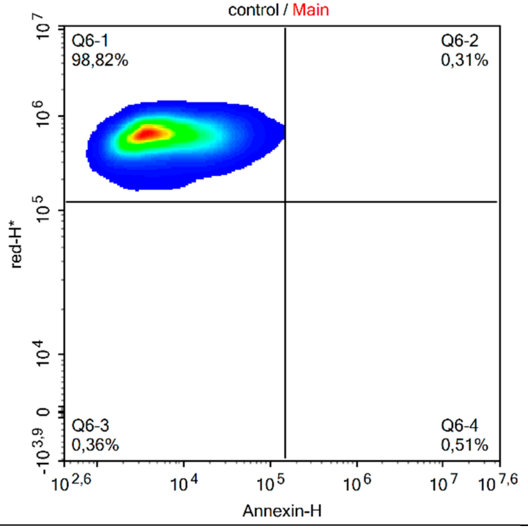

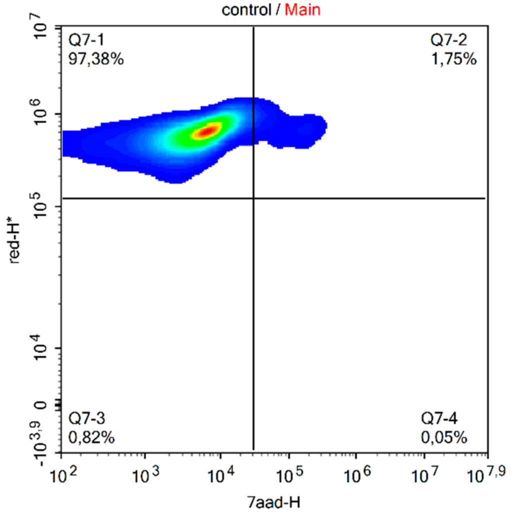

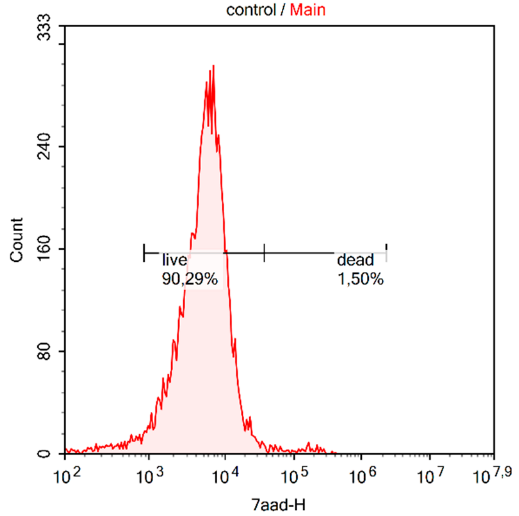

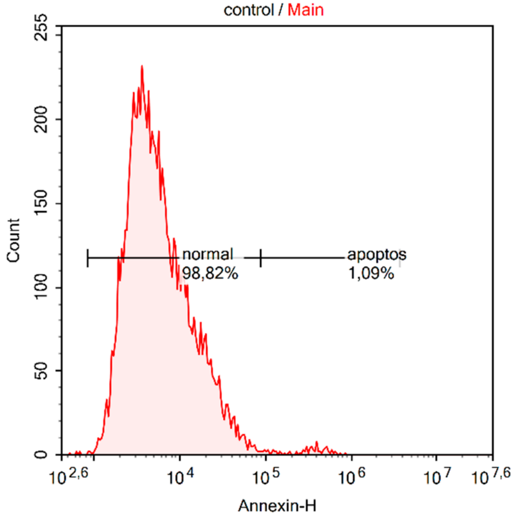

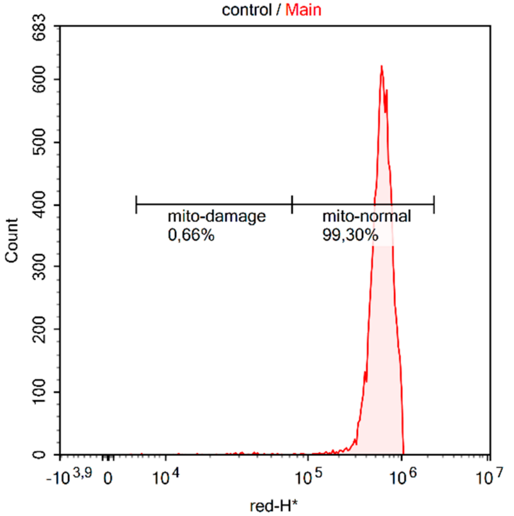

CCCP

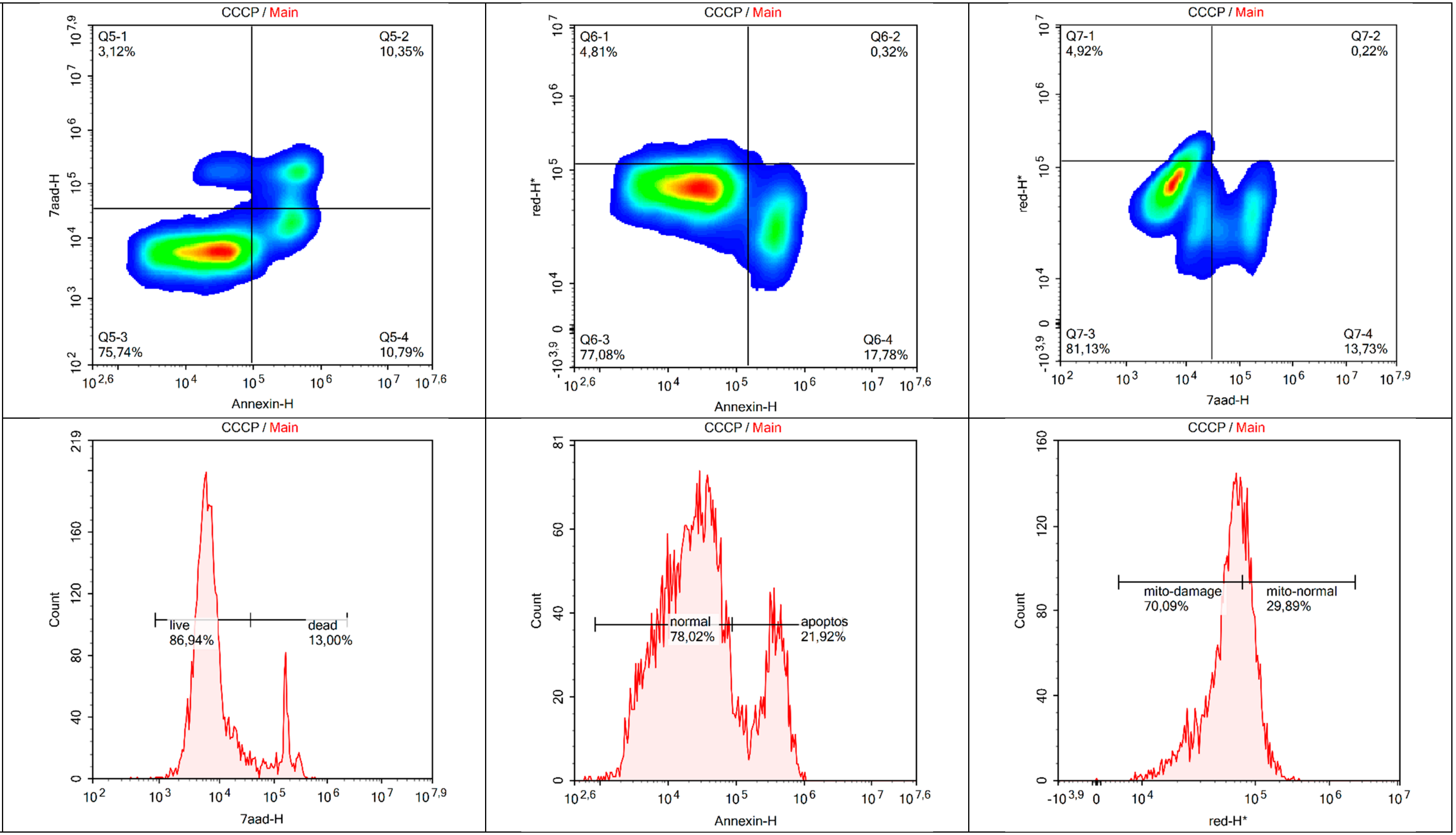
CCCP / Main
Q5-1
3,12%
Q5-2
10,35%
Q5-3
75,74%
Q5-4
10,79%
7aad-H
Annexin-H
CCCP / Main
Q6-1
4,81%
Q6-2
0,32%
Q6-3
77,08%
Q6-4
17,78%
red-H*
Annexin-H
CCCP / Main
Q7-1
4,92%
Q7-2
0,22%
Q7-3
81,13%
Q7-4
13,73%
red-H*
7aad-H
CCCP / Main
live
86,94%
dead
13,00%
Count
7aad-H
CCCP / Main
normal
78,02%
apoptos
21,92%
Count
Annexin-H
CCCP / Main
mito-damage
70,09%
mito-normal
29,89%
Count
red-H*

Str

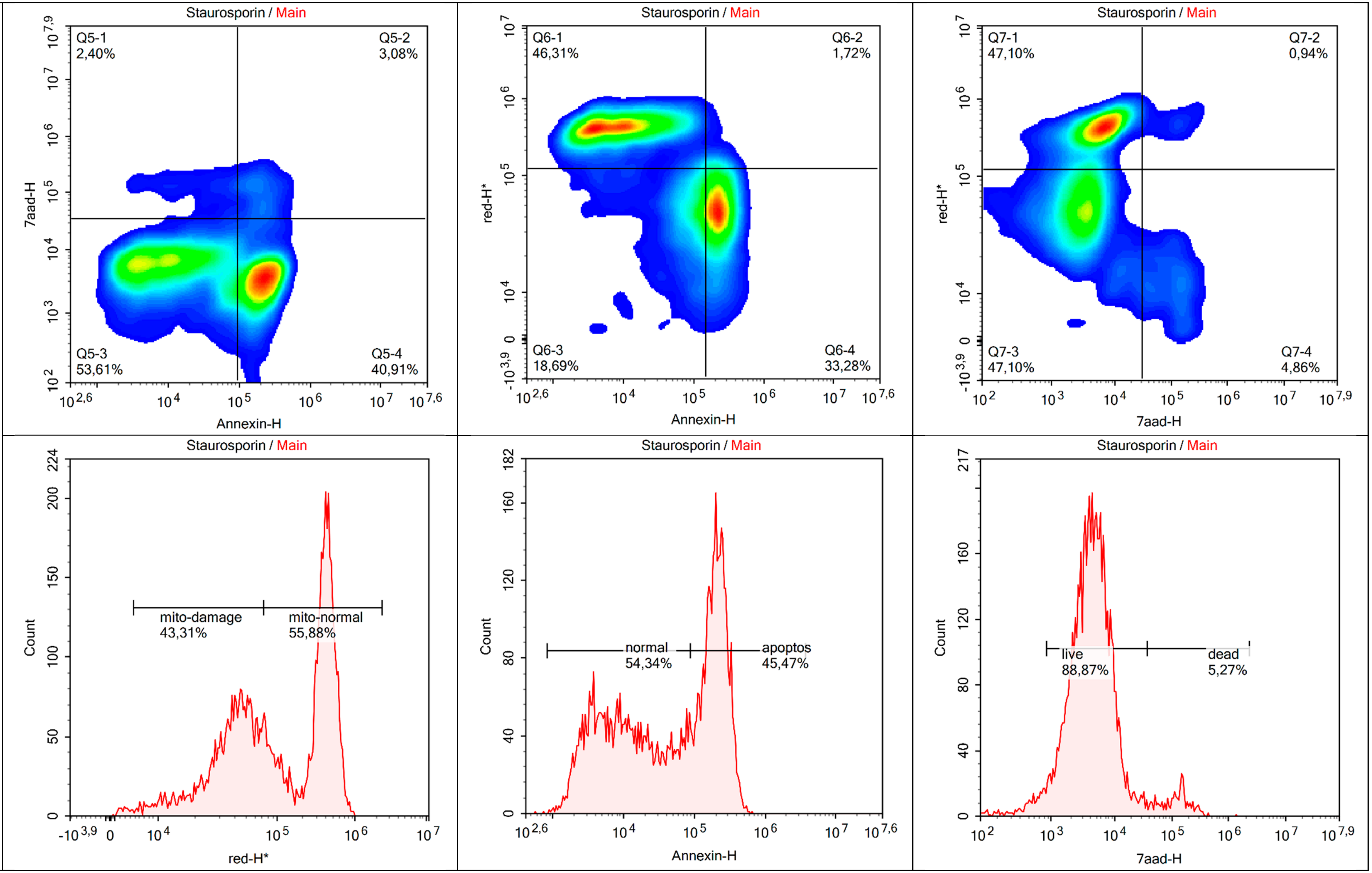

Staurosporin / Main
Q5-1
2,40%
Q5-2
3,08%
Q5-3
53,61%
Q5-4
40,91%
7aad-H
Annexin-H
Staurosporin / Main
Q6-1
46,31%
Q6-2
1,72%
Q6-3
18,69%
Q6-4
33,28%
red-H*
Annexin-H
Staurosporin / Main
Q7-1
47,10%
Q7-2
0,94%
Q7-3
47,10%
Q7-4
4,86%
red-H*
7aad-H
Staurosporin / Main
mito-damage
43,31%
mito-normal
55,88%
Count
red-H*
Staurosporin / Main
normal
54,34%
apoptos
45,47%
Count
Annexin-H
Staurosporin / Main
live
88,87%
dead
5,27%
Count
7aad-H

6d

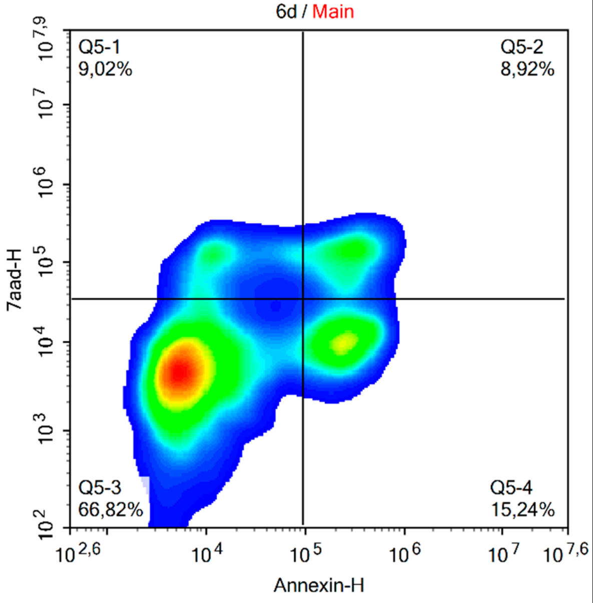

6d / Main
Q5-1
9,02%
Q5-2
8,92%
Q5-3
66,82%
Q5-4
15,24%
7aad-H
Annexin-H

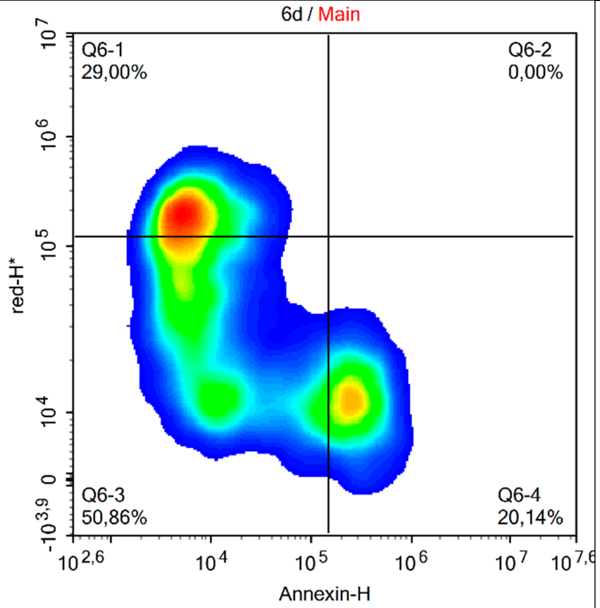

6d / Main
Q6-1
29,00%
Q6-2
0,00%
Q6-3
50,86%
Q6-4
20,14%
red-H*
Annexin-H

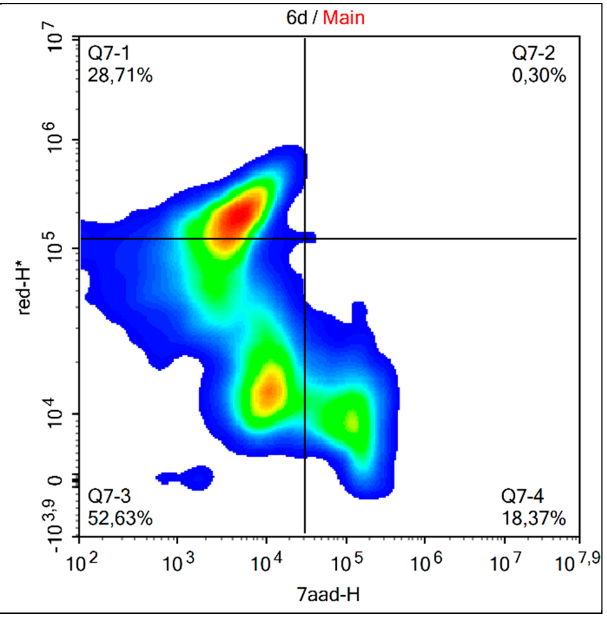

6d / Main
Q7-1
28,71%
Q7-2
0,30%
Q7-3
52,63%
Q7-4
18,37%
red-H*
7aad-H

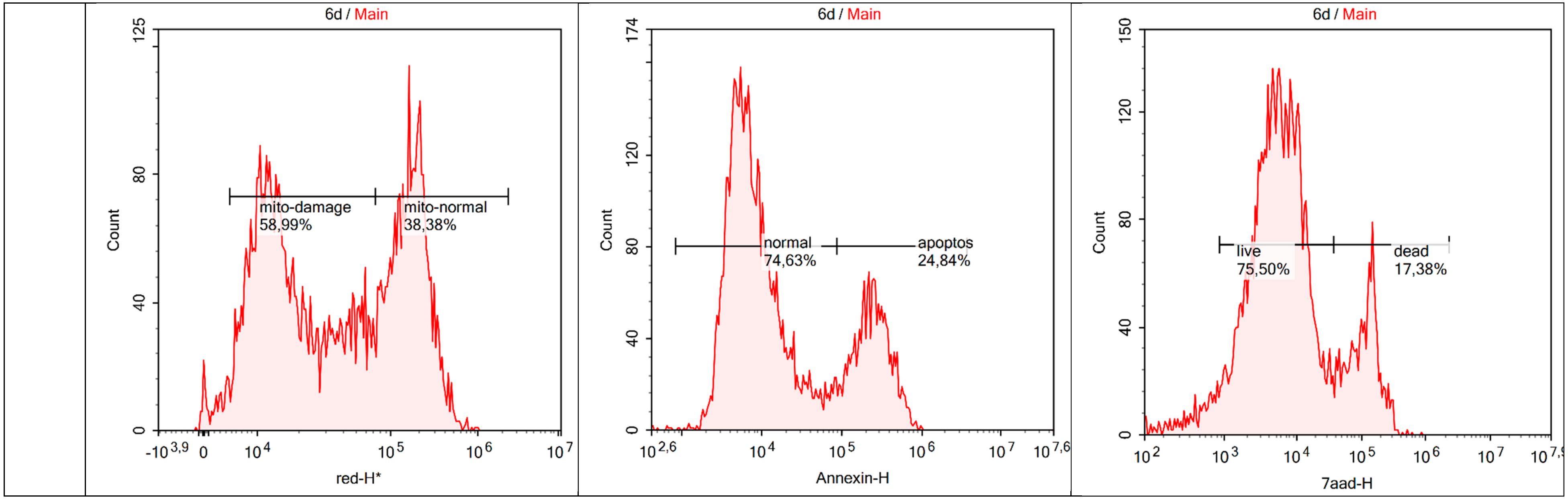
6d / Main
Count
red-H*
mito-damage
58,99%
mito-normal
38,38%
6d / Main
Count
Annexin-H
normal
74,63%
apoptos
24,84%
6d / Main
Count
7aad-H
live
75,50%
dead
17,38%

7d	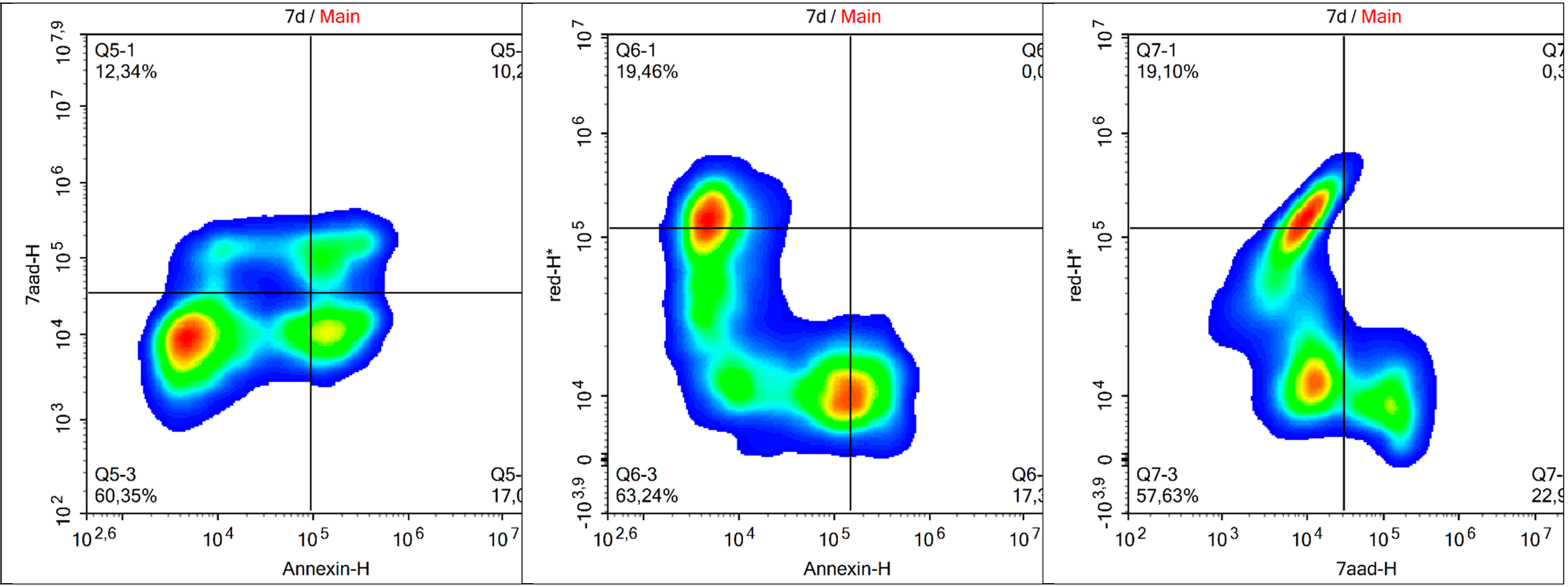

7d / Main
Q5-1
12,34%
Q5-
10,2
Q5-3
60,35%
Q5-
17,0
7aad-H
Annexin-H
7d / Main
Q6-1
19,46%
Q6
0,0
Q6-3
63,24%
Q6-
17,3
red-H*
Annexin-H
7d / Main
Q7-1
19,10%
Q7
0,3
Q7-3
57,63%
Q7-
22,9
red-H*
7aad-H

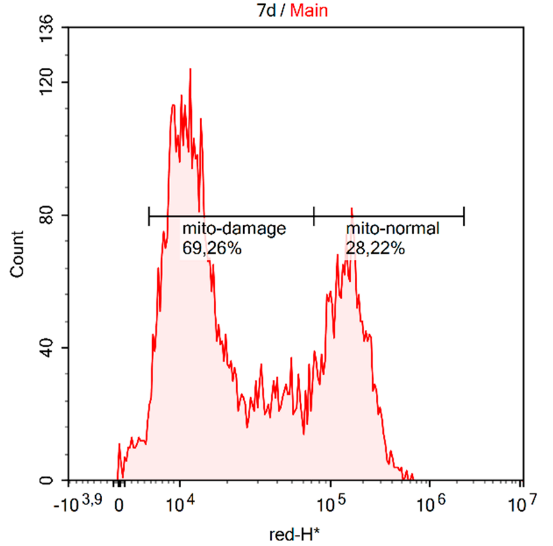
7d / Main
mito-damage
69,26%
mito-normal
28,22%
Count
red-H*

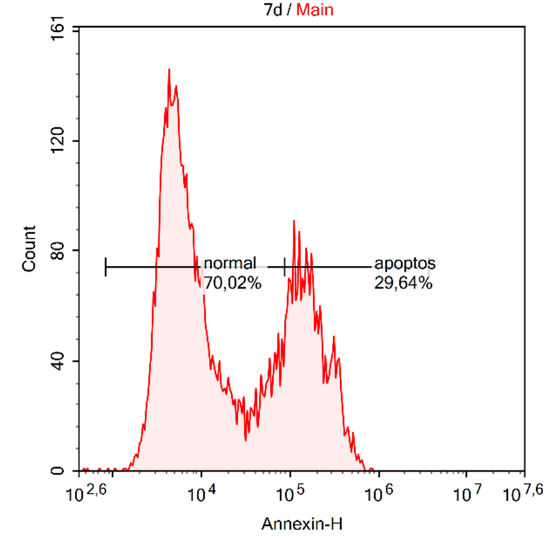
7d / Main
normal
70,02%
apoptos
29,64%
Count
Annexin-H

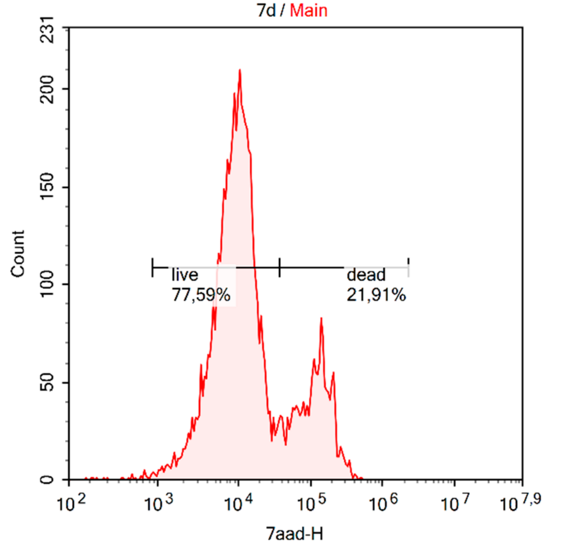
7d / Main
live
77,59%
dead
21,91%
Count
7aad-H

8d

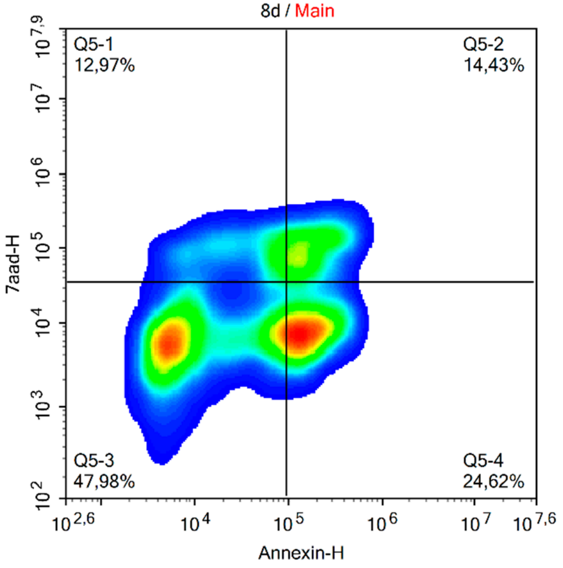
8d / Main
Q5-1
12,97%
Q5-2
14,43%
Q5-3
47,98%
Q5-4
24,62%
7aad-H
Annexin-H

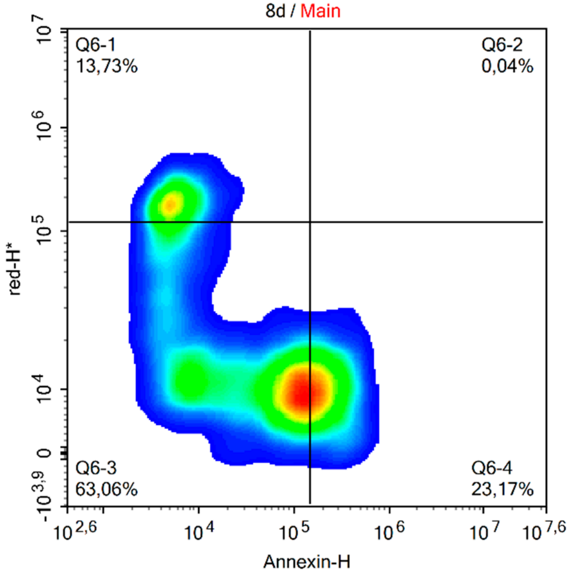
8d / Main
Q6-1
13,73%
Q6-2
0,04%
Q6-3
63,06%
Q6-4
23,17%
red-H*
Annexin-H

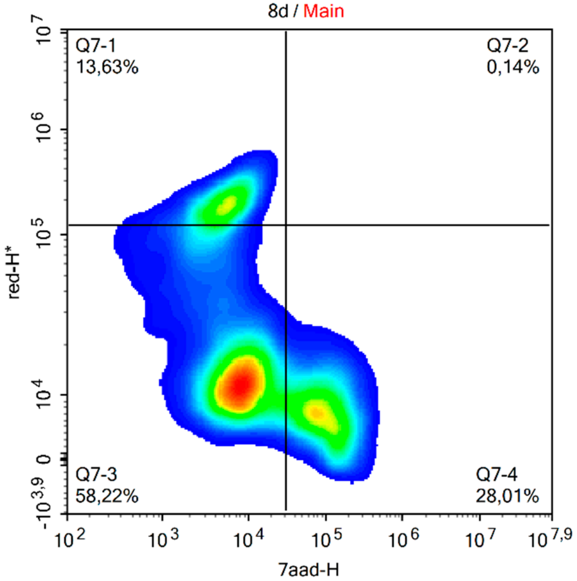
8d / Main
Q7-1
13,63%
Q7-2
0,14%
Q7-3
58,22%
Q7-4
28,01%
red-H*
7aad-H

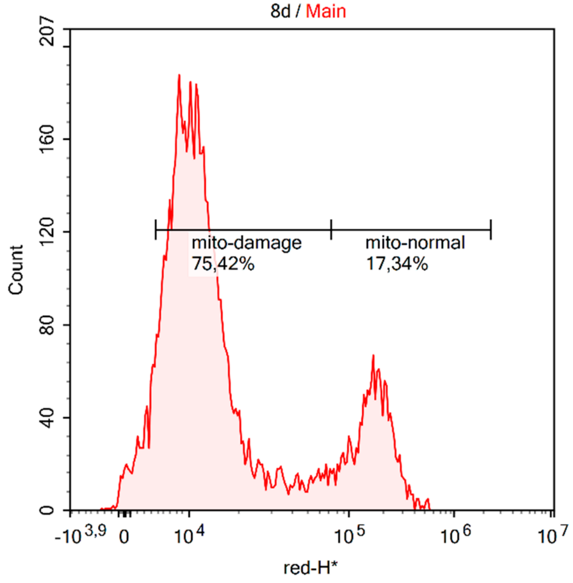
8d / Main
Count
red-H*
mito-damage
75,42%
mito-normal
17,34%

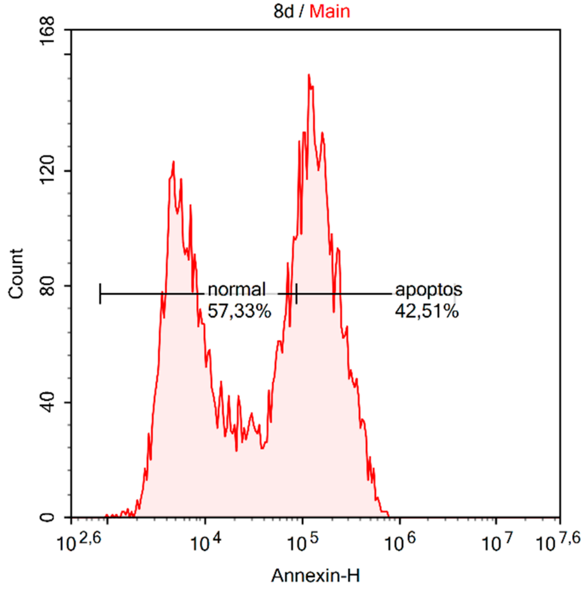
8d / Main
Count
Annexin-H
normal
57,33%
apoptos
42,51%

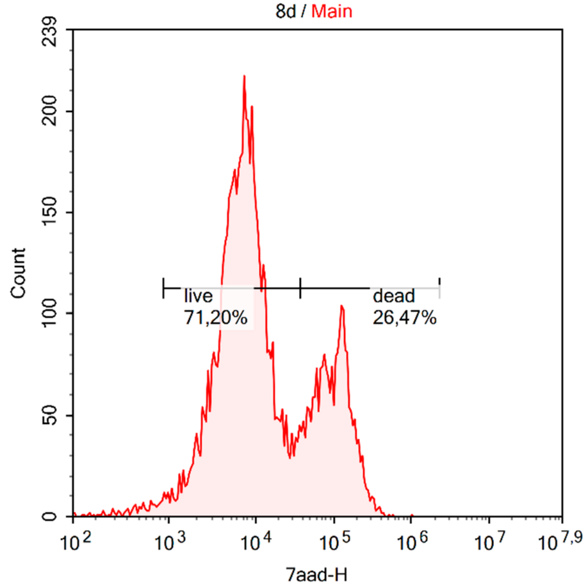
8d / Main
Count
7aad-H
live
71,20%
dead
26,47%

9d

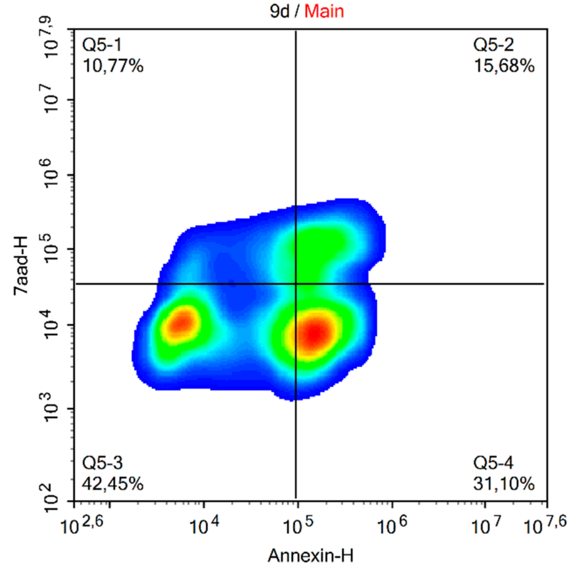
9d / Main
7aad-H
Annexin-H
Q5-1
10,77%
Q5-2
15,68%
Q5-3
42,45%
Q5-4
31,10%

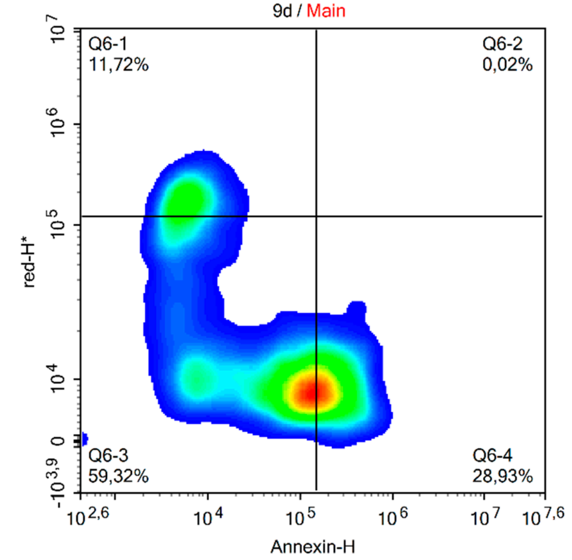
9d / Main
red-H*
Annexin-H
Q6-1
11,72%
Q6-2
0,02%
Q6-3
59,32%
Q6-4
28,93%

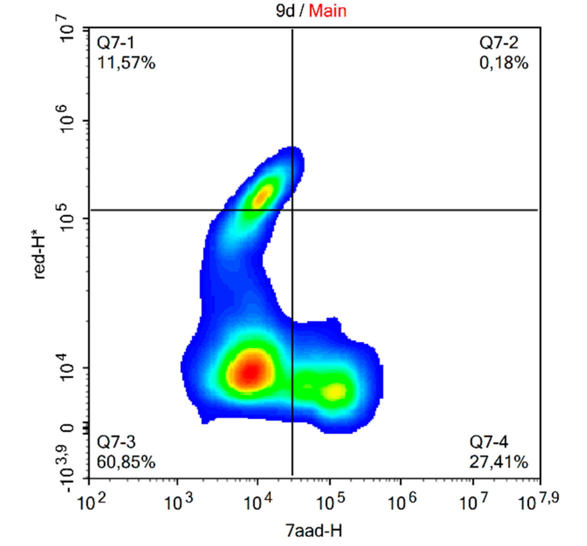
9d / Main
red-H*
7aad-H
Q7-1
11,57%
Q7-2
0,18%
Q7-3
60,85%
Q7-4
27,41%

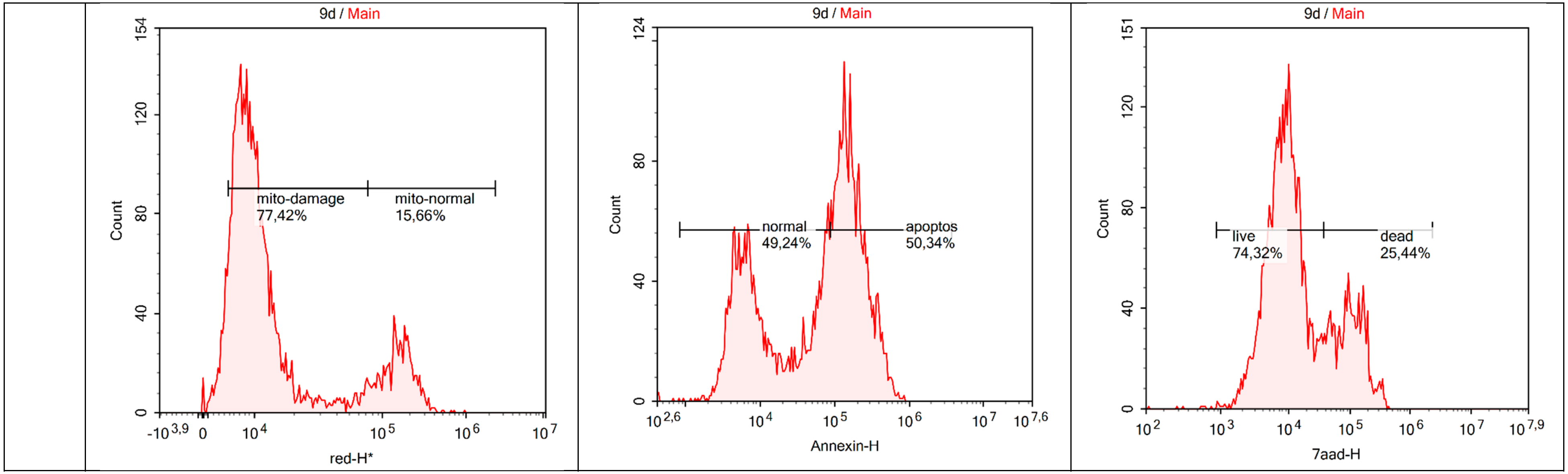
9d / Main
Count
mito-damage
77,42%
mito-normal
15,66%
red-H*
9d / Main
Count
normal
49,24%
apoptos
50,34%
Annexin-H
9d / Main
Count
live
74,32%
dead
25,44%
7aad-H
